# Diskursallianzen in der Migrationsdebatte? Politischer Parallelismus zwischen Medien und Parteien im Framing der Flucht- und Asylmigration im Jahr 2018

**DOI:** 10.1007/s11615-021-00324-z

**Published:** 2021-07-07

**Authors:** Raphael Kösters, Olaf Jandura, Ralph Weiß, Josef Schreiber

**Affiliations:** Institut für Sozialwissenschaften, Kommunikations- und Medienwissenschaft, Heinrich-Heine-Universität Düsseldorf, Düsseldorf, Deutschland

**Keywords:** Medien-Parteien-Parallelismus, Öffentlichkeitstheorie, Frames, Migration, Cleavages, Media–party parallelism, Theory of the public sphere, Framing, Migration, Cleavages

## Abstract

Die Studie untersucht, inwieweit Medien bei der Debatte um die Flucht- und Asylmigration die zentralen Deutungsmuster der Parteien wiedergeben und ob dabei Diskursallianzen zwischen einzelnen Medienangeboten und Parteien zum Vorschein kommen. Unser Beitrag widmet sich damit der öffentlichkeitstheoretisch relevanten Frage, ob bzw. welche Medien als Organe partikularer Standpunkte fungieren und welche durch eine breite und ausgewogene Abbildung konkurrierender Problemdeutungen die Grundlagen für eine unabhängige Meinungsbildung schaffen. Darüber hinaus prüfen wir, inwiefern Medien den Parteien eine faire Chance geben, dass ihre Positionen für das Staatsbürgerpublikum wahrnehmbar werden. Wir ermitteln das Ausmaß des politischen Parallelismus anhand einer Inhaltsanalyse von 18 Medienangeboten und von Dokumenten der sieben Bundestagsparteien. Auf der Ebene problembezogener Aussagen erfassen wir wertebezogene Deutungsmuster. So können wir die inhaltliche Substanz der vermittelten Positionen präzise nachzeichnen und Ähnlichkeiten von Medien und Parteien hinsichtlich ihrer Deutung des Streitthemas identifizieren. Ferner erlaubt unser Verfahren eine Verortung von Medien und Parteien im Raum politischer Positionen, der von Grundkonflikten (Cleavages) aufgespannt wird. Über diesen Rückgriff auf ein Konzept der politischen Soziologie können wir Nähe und Distanz von Medien und Parteien verdichtend beschreiben.

**Zusatzmaterial online:**

Zusätzliche Informationen sind in der Online-Version dieses Artikels (10.1007/s11615-021-00324-z) enthalten.

## Abstract (en)

Our study on political parallelism in Germany examines the extent to which media reflect the parties’ central frames in the debate on refugee and asylum migration. Thereby, we consider discursive alliances of individual media outlets and parties. In this context, we moreover address important questions of public sphere theory: Do the media function as organs of particular viewpoints, or do they set the ground for free opinion building by a broad and balanced representation of competing frames? In addition, we discuss the extent to which the media offer political parties a fair opportunity to make their positions observable to the public. Empirically, we analyze the extent of political parallelism based on a content analysis of 18 media outlets and official documents of the seven parties in the German Bundestag. At the level of issue-specific statements, we measure value-based frames. This approach enables us to describe the substance of mediated viewpoints concisely and to identify similarities between media and parties with regard to the framing of the issue. Furthermore, our method allows us to locate media and parties within the space of a basic political cleavage (integration vs. demarcation). Referring to a concept of political sociology enables us to describe the proximity and distance of media and parties in a condensed way.

## Einleitung

Öffentlichkeitstheoretische Ansätze erklären übereinstimmend *Pluralität* zu einem zentralen Qualitätsmerkmal der medialen Vermittlung gesellschaftlicher Streitthemen. Medien sollen die Bandbreite der verschiedenen Standpunkte in politischen Auseinandersetzungen möglichst vollständig und auf *ausgewogene* Art und Weise öffentlich wahrnehmbar machen. Damit unterstützen sie die freie Meinungsbildung der Bürger*innen und tragen zur öffentlichen Repräsentation der politisch heterogenen Gesellschaft bei (Weiß et al. 2016, S. 30–31; Ferree et al. 2002, S. 317–318). Herausgefordert wird dieses öffentlichkeitstheoretische Ideal durch Selektions- und Darstellungsregeln der Medien und deren Zusammenspiel mit den Kommunikationsstrategien politischer Akteure im Wettbewerb um mediale Aufmerksamkeit (Marschall und Weiß 2011, S. 21–22). In diesem Zusammenhang gilt der Fokus unseres Beitrages dem Phänomen des *politischen Parallelismus*, auch bezeichnet als *Medien-Parteien-Parallelismus*. Mancini (2015, S. 1) legt hierfür eine breite Definition vor. Politischer Parallelismus bezieht sich auf das Ausmaß, „to which media outlets tend to support and/or are linked to different cultures, ideas, and organizations that play some role within the political arena“. Unser Beitrag erörtert, inwieweit Medien als unabhängige Foren der gesellschaftlichen Auseinandersetzung fungieren oder ob sie als Alliierte politischer Parteien in Erscheinung treten. Dabei können zudem die Unterschiede in den *Resonanzchancen* für Parteipositionen in verschiedenen deutschen Medien beschrieben werden (Voltmer 1997, S. 172).

Pluralität meint – im Verständnis partizipativer Öffentlichkeitstheorien – mehr als das Spektrum der Positionen von Parteien. Zivilgesellschaftlichen Akteuren schreiben sie die Rolle zu, gesellschaftliche Problemwahrnehmungen auch und gerade dann gesellschaftlich verhandelbar zu machen, wenn sie im politischen Raum noch nicht bearbeitet werden. Gleichwohl kommt der öffentlichen Wahrnehmbarkeit der Parteipositionen eine herausgehobene Rolle zu. Denn Parteien fungieren als die parlamentarische Repräsentation der Politikpräferenzen von Teilen der Bevölkerung. Zudem stecken ihre Positionen den Bereich der Gestaltungsoptionen ab, die potenziell Geltung erlangen können. Daher konzentrieren wir uns darauf, ob und wie Medien die Vielfalt dieser Positionen ihrem Publikum zur Kenntnis bringen.

Wir *fragen* nach dem Ausmaß des – bislang nur selten geprüften – auf Medieninhalte bezogenen Parallelismus („content parallelism“, Artero 2015, S. 4). Wir untersuchen, inwieweit Medien die zentralen inhaltlichen Deutungsmuster von Parteien zu einem relevanten Streitthema wiedergeben und inwiefern sie dabei diskursive Allianzen mit einzelnen Parteien eingehen, indem sie deren Positionen bevorzugt übermitteln.^1^ Untersuchungsgegenstand ist die öffentliche Debatte um das Thema der *Flucht- und Asylmigration*. Hierbei handelt es sich um ein in den letzten Jahren gesellschaftsweit relevantes Konfliktthema. Im Falle eines solchen herausragenden, polarisierenden Streitthemas können Medien durch eine pluralistisch-ausgewogene Abbildung des Meinungsspektrums einen Beitrag zur politischen Integration der Gesellschaft leisten. Denn die Aufmerksamkeit für dasselbe Thema könnte die Chance erhöhen, als Rezipient*in vom Spektrum unterschiedlicher Sichtweisen in der Gesellschaft zu erfahren (Peters 2002, S. 33; Wessler 2002, S. 71–73). Plausibel erscheint demgegenüber aber auch die Annahme, dass die Allianzen von Medien und Parteien in dieser politisierten Situation besonders deutlich zum Vorschein kommen. Vor diesem Hintergrund ist es eine bislang offene empirische Frage, ob, und wenn ja, welche konkreten „Diskurskoalitionen“ (Schäfer 2008) sich bei dem Thema Flucht- und Asylmigration zwischen Medien und Parteien in Deutschland zeigen.

Der politische Parallelismus wird in der vorliegenden Studie auf Grundlage einer standardisierten Inhaltsanalyse von Beiträgen aus 18 deutschen Medien einerseits und von offiziellen Dokumenten (Pressemitteilungen, Redebeiträge zu Gesetzesdebatten und aktuellen Stunden, Regierungserklärungen sowie Positionspapiere) der sieben im deutschen Bundestag vertretenen Parteien andererseits untersucht. Die von den Parteien kommunizierten und in den Medien vermittelten politischen Standpunkte werden über wertebezogene Deutungsmuster erfasst. Diese liegen vor, „wenn Werte, die in der politischen Kultur sedimentiert sind, als übergeordnete Bezugsrahmen für Sachverhalte, Vorgänge oder Akteure fungieren“ (Scheufele et al. 2012, S. 432). Operationalisiert werden diese Deutungsmuster auf der Ebene einzelner problembezogener Aussagen (Standardargumente; Gerhards 2008). Mithilfe dieses Zugangs können wir die inhaltliche Substanz der Position der Parteien präzise bestimmen. Sodann lässt sich rekonstruieren, welche Problemdeutungen in der Berichterstattung der Medien vermittelt werden. Auf dieser Grundlage können wir nachzeichnen, wie vollständig oder selektiv einzelne Medien das Spektrum politischer Beurteilungen des Konfliktthemas Flucht- und Asylmigration darstellen. Diese Analyse geht über Studien hinaus, die im Kontext der Frage nach der Ausgewogenheit der Migrationsberichterstattung lediglich das Vorkommen von positiven und negativen Beurteilungen gegenüberstellen (z. B. Goedeke Tort et al. 2016; Maurer et al. 2019). Wir können demgegenüber nachzeichnen, mit welchen normativ aufgeladenen Deutungsmustern Parteien und Medien dem Thema inhaltlich Profil geben.

Dabei knüpfen wir an die Debatte um zentrale Wertekonflikte (Cleavages) an, die die Struktur politischer Auseinandersetzungen und die politische Kultur eines Landes prägen. Konkret beziehen wir Problemdeutungen von Parteien und Medien auf die Gegenüberstellung von integrativen und abgrenzenden Positionen zum Migrationsthema (Grande und Kriesi 2012, S. 6–8). Auf Grundlage unserer empirischen Auswertungen ist abzulesen, wo Medien und Parteien auf dieser Konfliktdimension zu verorten sind. Unser Ansatz verknüpft damit Überlegungen der Kommunikationswissenschaft mit denen der politischen Soziologie. Indem wir sowohl Medien als auch Parteien im Raum politischer Positionen verorten, der von Grundkonflikten aufgespannt wird, bringen wir *Diskursallianzen* zum Vorschein.

Der Beitrag ist folgendermaßen aufgebaut: Zunächst verorten unseren Beitrag in der vorliegenden Literatur zum politischen Parallelismus. Daran anschließend diskutieren wir die Bedeutung des politischen Parallelismus für das Funktionieren öffentlicher Kommunikation und leiten daraus unsere explorativen Fragestellungen ab. Der methodische Zugang über eine inhaltsanalytische Erfassung von normativ aufgeladenen Deutungsmustern, das analysierte Datenmaterial und unsere Auswertungsverfahren werden im dritten Abschnitt vorgestellt. Anschließend präsentieren wir die empirischen Ergebnisse. Im letzten Abschnitt liefern wir eine Zusammenfassung und – ausgehend von den Limitationen unserer Analysen – einen Ausblick auf mögliche Folgestudien.

## Politischer Parallelismus im Kontext der Funktionen öffentlicher Kommunikation für die Demokratie

### Das Konzept des politischen Parallelismus

Das Konzept des politischen Parallelismus geht zurück auf Seymour-Ure (1974). Ein „press-party-parallelism“ sei ihm zufolge gegeben, wenn eine Zeitung einer Partei in organisatorischer Hinsicht sowie auf ihre Ziele und Leserschaft bezogen nahesteht („closely linked to that party by organisation, loyalty to party goals and the partisanship of its readers“, S. 173). Aufbauend darauf prägten Hallin und Mancini (2004, S. 27) den allgemeineren Begriff des politischen Parallelismus, der berücksichtigt, dass Medienangebote mittlerweile eher selten mit einzelnen Parteien im Sinne einer „one-to-one connection“, wohl aber mit „general political tendencies“ in Verbindung stehen. Eine Erweiterung stellt zudem der Terminus „Media-Party-Parallelism“ nach van Kempen (2007) dar. Er drückt aus, dass Parallelstrukturen zwischen Medien und politischen Akteuren nicht nur im Zeitungsmarkt, sondern auch in anderen Mediengattungen existieren können.

Artero (2015, S. 3–5) fasst in einem Forschungsüberblick die unterschiedlichen Faktoren zusammen, die die Ausbildung von Parallelstrukturen begünstigen oder abschwächen können und die sich je nach Medien- und Politiksystem eines Landes unterscheiden (z. B. die Verankerung und Persistenz von gesellschaftlichen Cleavages, polarisierte Parteiensysteme, staatliche Interventionen im Mediensystem, mehr oder weniger politisierte Journalismuskulturen). Im internationalen Vergleich ist der Grad des politischen Parallelismus in *Deutschland* moderat (Brüggemann et al. 2014; van Kempen 2007; Hallin und Mancini 2004). Dennoch sind *redaktionelle Linien* wirksam, die als „langfristige Strukturmerkmale des deutschen Mediensystems“ (Zerback 2013, S. 193) die politische Ausrichtung der Berichterstattung einzelner Medien prägen (Scheufele und Engelmann 2014; Eilders 2004; Schönbach 1977).

Neben den Gründen des politischen Parallelismus widmet sich die Forschung der Frage, auf welcher *Ebene* und über welche *Indikatoren* Parallelstrukturen empirisch gemessen werden können. Auf organisatorischer Ebene ist von Interesse, inwieweit politische Akteure in Eigentumsverhältnisse sowie Managementfunktionen von Medienangeboten eingebunden sind. Auf der Publikumsebene kann geprüft werden, inwieweit sich unter der Nutzerschaft eines Mediums Anhänger*innen von bestimmten Parteien oder von politischen Grundhaltungen befinden. Auf Ebene der Medienakteur*innen wird unter anderem ermittelt, inwieweit sie sich an Professionalitätsstandards des Journalismus orientieren oder ob sich Politisierungstendenzen feststellen lassen (de Albuquerque 2013, S. 746–747; Artero 2015, S. 2–5; Mancini 2015; Pfetsch 2014). Hallin und Mancini (2004) haben eine wichtige Untersuchungsebene ergänzt, die Ebene der *Medieninhalte*. Unter Verweis auf Allern und Blach-Ørsten (2011) legt Artero (2015, S. 8) dar, dass Parallelitäten auf organisatorischer Ebene mittlerweile seltener geworden sind. Stattdessen seien Parallelstrukturen heutzutage auf inhaltlicher Ebene am ehesten manifest und messbar.

In der politischen Kommunikationsforschung existiert eine Vielzahl von Studien zu Konzepten, die dem politischen Parallelismus inhaltlich nahestehen. Zu diesen Konzepten zählen etwa die politische Ausgewogenheit, die Parteilichkeit bzw. „partisan bias“ oder die Synchronisierung von Berichterstattung und Kommentaren (Hopmann et al. 2011; Stalker 2013; Schönbach 2008). Der *empirische* Fokus dieser Studien liegt jedoch ausschließlich auf den publizistischen Leistungen der Medien, wobei in der Ergebnisinterpretation über Indikatoren wie Gewichtung von Themen, Präsenz und Valenz von Akteuren auf die Nähe zu Parteien geschlossen wird. Inhaltsanalytische Vergleiche mit Dokumenten aus dem politischen System werden abgesehen von vereinzelten Input-Output-Analysen und Strukturvergleichen kaum vorgenommen (Jandura und Leidecker 2015, S. 36–39).

Insgesamt überrascht es, dass empirische Studien, die sich explizit dem Ausmaß des Parallelismus auf inhaltlicher Ebene widmen, selten sind. Zwei Ausnahmen sollen an dieser Stelle referiert werden. Für Deutschland ist uns nur ein Forschungsprojekt bekannt, das inhaltsanalytisch vorgeht und dasselbe Kategoriensystem auf Medien- und Parteidokumente anwendet. Unter Rückgriff auf das ursprüngliche Konzept von Seymour-Ure (1974) untersucht Voltmer (1997, 1999) „ideologische Parallelstrukturen“ in der Berichterstattung von sieben Printmedien im Vergleich zu Pressemitteilungen der Parteien zum Wahlkampf 1990. Ähnlich unserer Studie erfasst Voltmer Medien- und Parteipositionen im Hinblick auf politische Grundkonflikte und differenziert dabei fünf Konfliktdimensionen (Wohlstand und Verteilung, Institutionelle und individuelle Autonomie, Kulturelle Identität, Gesellschaftliche Integration, Äußere Sicherheit) mit jeweils einer linken und einer rechten Position. Ihre Ergebnisse beziehen sich auf eine außergewöhnliche Situation der deutschen Geschichte, die Phase der deutschen Wiedervereinigung und die erste gesamtdeutsche Bundestagswahl. Für jede untersuchte Zeitung kann sie ein spezifisches „ideologisches Profil“ ausmachen und findet teils erwartete, teils überraschende, als „situationsspezifisch“ (Voltmer 1997, S. 173) einzuordnende Parallelstrukturen. Unsere Studie baut auf den Arbeiten von Voltmer auf und *erweitert* sie dadurch, dass das Mediensample deutlich ausgeweitet und die Berichterstattung über die Wahlkampfphase hinaus in den Blick genommen wird. Ferner werden neben Pressemitteilungen auch andere relevante Parteidokumente analysiert. Während Voltmer eine themenübergreifende Analyse durchführt, fokussieren wir auf ein ausgewähltes Issue. Denn das erlaubt eine besonders präzise Messung von inhaltlichen Parallelstrukturen.

Auch wenn das Konzept des politischen Parallelismus in der Studie von Roggeband und Vliegenthart (2007) nicht erwähnt wird, so ist der Beitrag für die vorliegende Studie von Relevanz. In ihrer Studie untersuchen sie im Längsschnitt (1995–2004) Parallelstrukturen beim Framing zum Thema Migration in der niederländischen Presse und der parlamentarischen Arena. Dabei zeigt sich, dass das Spektrum vielfältiger Frames in der parlamentarischen Arena nicht uneingeschränkt medial gespiegelt wird. In einer differenzierten Betrachtung wird deutlich, dass Qualitätsmedien vielfältiger berichten als Boulevardangebote – die Unterschiede sind allerdings nicht allzu groß. Darüber hinaus bestätigt sich die Wirksamkeit redaktioneller Linien für die inhaltliche Nähe von Parteien und Medienangeboten mit ähnlicher politischer Ausrichtung. Wie lassen sich solche Befunde öffentlichkeitstheoretisch einordnen?

### Öffentlichkeitstheoretische Standards und politischer Parallelismus

Das Framing von gesellschaftlichen Problemen und Prozessen ist so umstritten wie die politischen Positionen, mit denen es assoziiert ist. Soll beispielsweise die Migration unter dem Vorzeichen libertär-universalistischer oder national-völkischer, wohlfahrtsstaatlich-solidarischer oder marktliberaler Deutungen verstanden und praktisch behandelt werden? Zum Kampf der politischen Standpunkte gehört der *Deutungskampf* im Raum der öffentlichen Kommunikation (Kösters 2020, S. 19–22).

Das Phänomen des politischen Parallelismus besteht darin, dass Medien diesen Deutungskampf nicht allein wiedergeben, sondern in ihm als Organ einer partikularen politischen Position agieren und damit parteiisch in ihn eingreifen (Mancini 2015, S. 4). Diese Parteilichkeit kann auf verschiedene Weisen realisiert werden: durch die Akzentuierung von Facetten eines Themas, die einem politischen Standpunkt als Beleg seiner Richtigkeit dienen können („Instrumentelle Aktualisierung“, Kepplinger 1989), durch die Bevorzugung von Sprechern, die eine Position artikulieren, welche das Medium teilt („Opportune Zeugen“, Hagen 1992) oder durch das Favorisieren von Deutungsmustern, die auf die politische Weltanschauung des Mediums abgestimmt sind (Benson 2009; Roggeband und Vliegenthart 2007; Voltmer 1997).

Was bedeutet nun der politische Parallelismus für das Funktionieren öffentlicher Kommunikation? Indem Medien sich als diskursive Plattform eines politischen Lagers oder Milieus anbieten, schaffen sie eine Grundlage für die Partizipation von Bürger*innen an der Politik. Denn sie ermöglichen die Fortbildung einer weltanschaulich fundierten Haltung zur Meinung in Fragen der Tagespolitik. Zudem kann das Milieu seine Auffassung legitimer Politik im öffentlichen Raum artikuliert finden. Wenn unterstellt werden kann, dass sich für divergierende politische Milieus im Spektrum der Medien jeweils ein Organ ihrer Weltanschauung findet, gibt es dann bei dieser Vielfalt im Sinne des Modells des *Außenpluralismus* (Zerback 2013, S. 105–109) überhaupt ein Problem für die Rolle öffentlicher Kommunikation in einer Demokratie?

Öffentlichkeitstheorien leiten Standards für das Funktionieren öffentlicher Kommunikation aus Prinzipien der Demokratie selbst ab – wie Volkssouveränität, Freiheit und Gleichheit. Sie nehmen daher auch die Kontroverse in der Demokratietheorie darüber auf, was zu den Wesensbestimmungen einer Demokratie gehört (Ferree et al. 2002; Jandura und Friedrich 2014). Zu den unstrittigen Bestimmungen gehört jedoch, dass Demokratien ein *Forum* brauchen, auf dem für alle wahrnehmbar und unter Beteiligung aller für eine Gesellschaft relevanten Standpunkte der Streit um richtige Politik ausgetragen werden kann – also der Konflikt darum, welche Probleme Vorrang bekommen müssen und welche Maßnahmen zur Problemlösung legitim sind.

Ein solches Forum existiert nicht bereits durch das Nebeneinander je parteilicher Weltdeutungen, wenn die betreffenden Medienarenen konkurrierende Positionen nicht oder allenfalls in der Form der Diskreditierung zum Vorschein kommen lassen. Ein gesellschaftliches Forum entsteht erst, wenn Medien sich zu einer *offenen Plattform* machen, auf der die jeweiligen Lager wahrnehmen können, was für die Angehörigen einer anderen Weltauffassung wichtig ist und welche Gestaltungsoptionen sie favorisieren. Erst dadurch entsteht die Chance, sich lagerübergreifend darüber zu verständigen, welche Probleme beachtet und bearbeitet werden sollten. Diese milieuübergreifende *Themenkonsonanz* ist eine elementare Voraussetzung für einen öffentlich ausgetragenen Streit, aus dem Legitimität für die am Ende gültig gemachte Entscheidungsoption erwachsen kann (Weiß und Jandura 2017, S. 18–19).

Zur Funktion als Forum gehört darüber hinaus, dass die konkurrierenden Positionen in ihren sachlichen und normativen Geltungsansprüchen wahrnehmbar werden. Die Darstellung von Positionen mit ihren Begründungen schafft erst die Grundlage für eine Auseinandersetzung, die als Streit um das bessere Argument ausgetragen wird. Nur ein deliberativ ausgetragener Konflikt setzt das Rationalitätspotenzial öffentlicher Debatte frei und sorgt zugleich für die Zivilisierung der Konfliktaustragung (Habermas 2006, S. 413; Imhof 2011, S. 104–105). Aus der Perspektive des Individuums betrachtet geben erst in diesem Sinn *binnenplurale* Medien Bürger*innen die Chance, eine eigene Position in der Abwägung zwischen den vorgestellten Politikalternativen zu bilden. Der Pluralismus als Merkmal der publizistischen Leistung ist die Voraussetzung individueller Freiheit – der Freiheit von der Ohnmacht des Unvermögens zur Beurteilung relevanter gesellschaftlicher Probleme oder der unaufgeklärten Bindung an ein politisches Lager (Dahl 1989, S. 307; Donsbach 2008, S. 196–199).

Das sind Gründe für *Ausgewogenheit *und* Fairness* als professionelle Standards des Journalismus bei der Berichterstattung über gesellschaftliche Konflikte. Der politische Parallelismus steht zu diesen Standards in einem spannungsvollen Verhältnis. Das Problem wird dort sinnfällig, wo das Auseinandertreten der Themenhorizonte und die Einseitigkeit der Problemdeutung zu einer *Polarisierung* fortgetrieben werden, in der sich die politischen Lager in ihre Echoräume zurückziehen und untereinander weder diskursfähig noch kompromissbereit sind (Bennett und Iyengar 2008, S. 720–726).

Vor diesem Hintergrund stellen sich für unsere Studie mit Bezug auf die mediale Berichterstattung folgende *forschungsleitende Fragen*: Wie ist es um die Binnenpluralität der Berichterstattung über die Positionen der Parteien beim Konfliktthema der Flucht- und Asylmigration bestellt? Gibt es Medien, die Lagerjournalismus betreiben? Was bedeutet das für die Chance der Nutzer, konkurrierende Standpunkte im Kontext ihrer Begründungen wahrzunehmen? Welche Medien erfüllen demgegenüber die Forumsfunktion öffentlicher Kommunikation? Zur Beantwortung dieser Fragen inspizieren wir die mediale Repräsentation von Frames, mit denen die konkurrierenden Parteien ihre Position in der Sache artikulieren. Da es bisher nur sehr wenige Studien zum politischen Parallelismus auf der Ebene der Medieninhalte gibt, besteht unser Anliegen auch darin, einen Weg aufzuzeigen, wie die Parallelität der Problemdeutungen zum Vorschein gebracht werden kann.

## Methodisches Vorgehen

Anhand einer standardisierten Inhaltsanalyse von 18 Medienangeboten und offiziellen Dokumenten der sieben im deutschen Bundestag vertretenen Parteien (Pressemitteilungen, Redebeiträge zu Gesetzesdebatten und aktuellen Stunden, Regierungserklärungen sowie Positionspapiere) ermitteln wir das Ausmaß des politischen Parallelismus in der öffentlichen Debatte um das Issue Flucht- und Asylmigration. Das *Mediensample* enthält Informationsangebote unterschiedlichen Typs (Qualitäts- und Boulevardjournalismus, öffentlich-rechtlicher und privater Rundfunk, Regionaljournalismus, reiner Online-Journalismus) und unterschiedlicher Gattungen (Print, Rundfunk, Online), die in den Informationsrepertoires verschiedener Bevölkerungssegmente von Relevanz sind (Hasebrink und Hölig 2020). Zudem wird mit Blick auf die redaktionellen Linien das gesamte politisch-publizistische Spektrum abgedeckt (siehe ausführlich Kösters 2020; Kap. 4.2).

Der *Untersuchungszeitraum* umfasst die Monate März bis Juli 2018. Abweichend davon wurden Positionspapiere der Parteien, die nicht in diesem Zeitraum veröffentlicht wurden, aber einen thematischen Bezug aufweisen, ebenfalls in das Untersuchungsmaterial aufgenommen. Positionspapiere stellen im Vergleich zu den eher tagesaktuellen Pressemitteilungen und Bundestagsreden eine längerfristig gültige politische Positionierung dar. Sie werden zudem durch die Parteien und Fraktionen selbst veröffentlicht und drücken so noch mehr eine kollektive Position aus als die beiden anderen Texttypen, die zumeist von Einzelpolitiker*innen in die politische Debatte eingebracht werden. Die Länge des Untersuchungszeitraums soll sicherstellen, dass die Breite von thematischen Facetten der öffentlichen Debatte über Flucht- und Asylmigration in die Analyse einfließt.

Im Rahmen der *Stichprobenbildung* wurde für die Parteidokumente eine Vollerhebung durchgeführt. Aufgrund der hohen Zahl thematisch einschlägiger Beiträge musste für das Mediensample eine Stichprobe gezogen werden. Als Verfahren haben wir uns für eine disproportional geschichtete Stichprobe (mit Medienangeboten als Schichten) entschieden, das präzisere Schätzungen der analysierten Parameter als einfache Zufallsstichproben erlaubt. Pro Medienangebot beträgt der Stichprobenfehler maximal 5 % (Vertrauensintervall von 95 %). Aufgrund der hohen Anzahl von Beiträgen und aus forschungsökonomischen Gründen haben wir den maximal akzeptierten Stichprobenfehler für die Online-Medien auf 10 % gesetzt. Im Ergebnis ging beispielsweise die Süddeutsche Zeitung mit 208 Beiträgen und über 300 Standardargumenten und ein Online-Angebot wie tagesschau.de mit 79 Beiträgen inklusive 117 Standardargumenten in unser Sample ein.^2^ Insgesamt analysierten fünf Codierer 2197 Medienbeiträge inklusive 3159 problembezogene Deutungen in Form von Standardargumenten und 1059 Dokumente der Parteien, die 959 problembezogene Deutungen enthalten (siehe für das Parteien- und Mediensample samt Fallzahlen die Tabellen 1 und 2 im Online-Anhang).

### Messungen

Um politische Standpunkte zum Thema Flucht- und Asylmigration inhaltsanalytisch zu *operationalisieren*, stützen wir uns auf öffentlichkeitssoziologische Framing-Ansätze. In Anlehnung an Gerhards (2008) operationalisieren wir Frames auf der Ebene problembezogener Aussagen, die im Diskurs von öffentlichen Sprechern vorgebracht werden. Diese Aussagen sind Standardargumente, die am Thema der Fluchtmigration einen Aspekt als zentrales Problem fokussieren und mit der Problemdefinition auch eine Deutung von Problemursachen und erforderlichen Maßnahmen der Problemlösung umfassen. Die Art der Problemdeutung macht sie zu „Aussagen, die sich auf Werte und Grundsatzfragen beziehen“ (Gerhards 1996, S. 90). In diesem Sinne erfassen wir Frames als normative, aufgeladene Deutungsmuster. Im Rahmen der Entwicklung eines inhaltsanalytischen Kategoriensystems wurden insgesamt 27 Frames aus der theoretischen und empirischen Literatur deduktiv abgeleitet. Diese Frames decken das breite Spektrum wertebezogener Perspektiven auf das Migrationsthema ab. Sie enthalten unter anderem Deutungen, die die ethische Verpflichtung zur Unterstützung von Flüchtlingen unterstreichen, Standpunkte zur kulturellen und wirtschaftlichen Dimension der Migration sowie politische und administrative Ansätze zur Problemlösung.^3^

**Table 1:** 

Konfliktpol	Kurzbezeichnung Frames
Integration	*Befürwortung* von:
	No Borders
	Gesellschaftlicher Kosmopolitismus
	Humanitarismus
	Barmherzigkeit (religiös)
	Multikulturalismus
	Toleranz (religiös)
	Republikanismus
	Innere Sicherheit: präventiv/integrativ
	(Internationale Kooperation)
	Linke Elitenkritik
	Politische Kultur nach rechts
	*Ablehnung abgrenzender Deutungen*
Abgrenzung	*Befürwortung* von:
	Assimilation
	Utilitarismus Aufnahmegesellschaft
	Anti-Egalitarismus und Ethnopluralismus
	Innere Sicherheit: Law-and-Order
	Ökonomisch nützliche Migrationstypen
	Wohlfahrtschauvinismus
	(Nationalstaatliche Souveränität)
	Effektivität des Regierens
	Moderat autoritäre Elitenkritik
	Extrem autoritäre Elitenkritik
	Politische Kultur nach links
	*Ablehnung integrativer Deutungen*

Die Kursivierung in der Tabelle gibt die Valenz der zugeordneten Frames an

Zusätzlich zum Vorkommen von Standardargumenten in der Berichterstattung sowie in den Dokumenten politischer Parteien erfassen wir auch die Valenz der Frames. Es macht nämlich einen Unterschied, ob beispielsweise die Darstellung von Migration als Gefahr für die innere Ordnung von einem Sprecher befürwortet, abgelehnt oder ambivalent bewertet wird. Die Messung des bloßen Vorkommens eines Frames verdeckt wichtige Unterschiede in der ideologischen Positionierung der jeweiligen Akteure.

In Anlehnung an Höglinger et al. (2012) werden die im Diskurs prominentesten Frames samt ihrer Valenz Positionen im politischen Konflikt zwischen Integration und Abgrenzung zugeordnet (siehe Tab. 1). Hierbei handelt es sich um einen politischen Grundkonflikt, einen *Cleavage*, der politische Debatten in Deutschland maßgeblich beeinflusst. Im weitesten Sinne bezieht sich der integrative Pol auf eine „universalist, multiculturalist or cosmopolitan position“, während den abgrenzenden Pol eine Position „in favour of protecting the national culture and citizenship in its civic, political and social sense“ (Kriesi et al. 2008, S. 11) kennzeichnet. Die Zuordnung der Standardargumente erfolgt aufgrund der inhaltlichen Passung zu dieser Definition der Konfliktpole. Die Konzeptualisierung des Grundkonflikts zwischen Integration und Abgrenzung baut auf die unter anderem von Kitschelt (1994) vertretene Vorstellung eines zweidimensionalen politischen Raums mit einer ökonomischen und einer kulturellen Konfliktdimension auf. In ihrer „Einbettungshypothese“ argumentieren Kriesi und Kollegen (2008, S. 12–14), dass diese zweidimensionale Struktur noch immer sinnvoll sei, während sich ihre Bedeutung durch den Bezug auf die Relevanz von Globalisierungsfragen verändert hat. Die hier vorgestellten, knappen Definitionen der Konfliktpole beziehen sich nur auf die kulturelle Dimension der Cleavages.^4^

Indem wir die medial präsentierten und in politischen Dokumenten vorgebrachten Argumente den Konfliktpositionen zwischen Integration und Abgrenzung zuordnen, können wir beschreiben, welche Position die Medien und die politischen Akteure in dem politischen Grundkonflikt einnehmen. Dies erlaubt zudem eine erweiterte Bestimmung der politischen Ausgewogenheit der medialen Berichterstattung. Zusammengenommen sind Konfliktpositionen und Standardargumente hierarchisch geordnet. Standardargumente (Frames) erfassen wir präzise auf der Ebene einzelner Aussagen, mit denen eine Haltung im Konflikt definiert wird. Konfliktpositionen abstrahieren von den einzelnen Aussagen und ordnen sie den Polen des Cleavages Integration versus Abgrenzung zu.

Insgesamt ist die *Reliabilität* der Codierung zufriedenstellend. Im Rahmen der Reliabilitätsprüfung haben wir uns für ein zweistufiges Verfahren entschieden. Für die Berechnung der Identifikationsreliabilität, d. h. die konsistente Identifikation von Standardargumenten in den Texten, haben wir das Übereinstimmungsmaß nach Holsti herangezogen. Für dichotome Variablen ist das Reliabilitätsmaß nach Krippendorff demgegenüber nur bedingt geeignet (für eine Diskussion siehe Polownikow 2017, S. 136–138). Der Übereinstimmungsgrad der Identifikationsreliabilität beträgt durchschnittlich 70 %. Die Reliabilität der weiteren Komponenten des Codierprogramms liegt bei einem Krippendorff-Alpha-Wert von 0,9 (Sprecher), 0,9 (Deutungsmuster) und 0,8 (Valenz).

### Analyselogik

Ziel unserer Analysen ist es, festzustellen, inwieweit die Behandlung des Themas Flucht- und Asylmigration in einzelnen Medien das gesamte Spektrum der Deutungsmuster der Parteien wiedergibt oder aber das Deutungsrepertoire einzelner Parteien privilegiert. Es geht daher stets um den Vergleich der Problemdeutungen, die in den Medien aufscheinen, mit denen der Parteien. Dieser Vergleich kann auf der Grundlage der abgestuften Klassifikation der Inhalte auf mehreren Ebenen und anhand mehrerer Auswertungsvarianten durchgeführt werden.

Zunächst prüfen wir, welche Deutungsmuster samt ihrer Valenz besonders häufig in einzelnen Medien und in den Parteidokumenten vorkommen. Diese *Deutungsprofile* geben erste Hinweise auf Ähnlichkeiten zwischen Medien und Parteien und die jeweilige inhaltliche Konturierung des Themas. Bei der Darstellung konzentrieren wir uns auf die fünf häufigsten Frames. Zum Zwecke der Übersichtlichkeit fokussieren wir uns bei den medialen Deutungsprofilen auf die Printmedien in unserem Sample.^5^ Diese fungieren weiterhin als *Leitmedien* der deutschen Medienlandschaft und prägen den öffentlichen Diskurs in besonderer Weise (Jarren und Vogel 2011). Mit der tageszeitung (taz), junge Welt und Junge Freiheit werden zudem „alternative“ Medien mit Relevanz für entsprechende Publikumssegmente berücksichtigt. Damit wird bei unserer Selektion der Printmedien auch die richtungspolitische Breite des publizistischen Spektrums abgedeckt.

Ein verdichtetes Bild der Ähnlichkeit ergibt sich, wenn geprüft wird, inwieweit Medien Deutungen dasselbe Gewicht geben wie einzelne Parteien. Darüber geben *Rangkorrelationen* Auskunft – allerdings ohne über den Rangplatz hinaus die relative Häufigkeit einzelner Standpunkte zu berücksichtigen. Dennoch dient eine entsprechende Analyse als hilfreicher Anhaltspunkt. Wir konzentrieren uns hierbei auf eine Auswahl von Deutungsmustern: Die Rangordnungen der Parteien und der Medien beziehen sich auf die 18 innerhalb des gesamten Parteiensamples prominentesten Deutungsmuster. Einzelne Deutungsmuster, die in Medien prominent vorkommen, spielen zum Teil aufseiten der Parteien eine geringere Rolle und werden nicht in die Analyse miteinbezogen. Als Rangkorrelationskoeffizient nutzen wir *Kendall-tau‑b*, der für quadratische Kontingenztabellen geeignet ist und mit dem die im Material vorliegenden Rangbindungen berücksichtigt werden können (Cleff 2012, S. 119–125; Müller-Benedict 2011).

Auf der von den einzelnen Frames abstrahierten Ebene der *Positionierung in der grundlegenden Konfliktdimension Integration versus Abgrenzung* kann geprüft werden, welchen Parteien die einzelnen Medien in ihrer Positionierung ähneln. Hierzu wird in einer *Kontingenztabelle* der Anteil von integrativen und abgrenzenden Konfliktpositionen pro Medium bzw. Partei gegenübergestellt. Vorab wurden Frames samt ihrer Valenz den Konfliktpolen wie oben beschrieben zugeordnet (Tab. 1).^6^

Darüber hinaus lässt sich prüfen, inwieweit Medien das Spektrum der Parteipositionen widerspiegeln oder nicht. Dafür nutzen wir den *Reflective-Diversity(RD)-Index* nach van Cuilenburg (2007). Der RD-Index gibt an, inwiefern eine empirische Referenznorm, das meint hier Parteipositionen in der Konfliktdimension zwischen Integration und Abgrenzung, medial abgebildet wird. Ausgangspunkt der Distanzberechnung zwischen Medien und Parteien sind die Mittelwerte von Medienangeboten und Parteien für das Vorkommen von integrativen (−1) und abgrenzenden (+1) Deutungsmustern. Rechnerisch wird dann die Differenz der beiden Mittelwerte durch zwei geteilt und von der Zahl eins abgezogen. Der resultierende Kennwert umspannt theoretisch den Wertebereich zwischen null (minimale Übereinstimmung von Parteien und Medien) und eins (perfekte Übereinstimmung). Diese Auswertungsvariante liefert ein kompaktes Bild über die Existenz von diskursiven Allianzen und Distanzen im Migrationsdiskurs.

Vor der Berechnung des RD-Indexes muss noch ein Maßstab für die *angemessene* Repräsentation der Position der verschiedenen Parteien festgelegt werden. Wie stark sollten die konkurrierenden Parteien mit ihren jeweiligen Positionen berücksichtigt werden? Welche Erwartung an die Darstellungsleistung der Medien lässt sich begründen? Im Kontext der Forschung zur Akteursvielfalt in der Medienberichterstattung wird diese Frage thematisiert. Dabei steht im Mittelpunkt, ob die Massenmedien im ausreichenden Maße offen für Minderheitenpositionen sind oder ob sie durch ihre auf das politische Establishment zentrierte Berichterstattung den Status quo im politischen System aufrechterhalten (Bonfadelli 2008, S. 7). Als Kompromiss theoretischer Argumente für und gegen eine stärkere Berücksichtigung minoritärer Positionen hat sich im politischen System der Bundesrepublik das *Prinzip der abgestuften Chancengleichheit* etabliert (Jandura 2011; Jandura et al. 2019). Hierbei werden weder alle Parteien einheitlich behandelt noch einzig der Stimmenproporz als Maßstab herangezogen. Basierend auf einer Einigung der Parteifraktionen im Parlament zu den Redezeiten und auf Gerichtsurteilen zur Vergabe staatlicher Leistungen (z. B. Fraktionsfinanzierung, Vergabe von kostenlosen Wahlwerbespots im Fernsehen) wurde ein Verfahren etabliert, das zum einen minoritären Positionen Sichtbarkeit verleiht und zum anderen den Wählern zu erkennen gibt, welche Parteien ein größeres Gewicht im politischen System haben. Vor diesem Hintergrund werden bei der Berechnung des RD-Indexes die Parteien entsprechend des Prinzips der abgestuften Chancengleichheit gewichtet. Die Regierungsparteien CDU und SPD werden doppelt, alle anderen Parteien einfach gewichtet (Verhältnis 2:1).

## Empirische Ergebnisse

### Deutungsprofile der Parteien

Anhand der Deutungsprofile illustrieren wir, auf welche Weise die Parteien ihre Position zum Streitthema Migration definieren. Daran kann zudem abgelesen werden, wie selektiv die Parteien ihre Position anlegen, d. h. inwiefern sie sich auf wenige Frames konzentrieren (Tab. 3 im Online-Anhang). Die Befunde lassen sich in folgenden Feststellungen zusammenfassen.

- *Parteien gesamt*: Innerhalb der Parteienlandschaft kommen drei Frames vergleichsweise häufig vor. Dazu zählen das Deutungsmuster, das „Internationale Kooperation“ zum Schlüssel für die Problemlösung ernennt, sowie integrative und abgrenzende Varianten der Elitenkritik („Linke Elitenkritik“, die mangelnde Anstrengungen zur Versorgung und Integration der Geflüchteten anprangert, „Moderat autoritäre Elitenkritik“, die den Verantwortlichen Fehler und Versagen bei der Abwehr der Probleme, die aus der Migration erwachsen, vorwirft). Formen der Elitenkritik – inklusive der an vierter Stelle der zusammengefassten Top-Deutungen rangierenden „extrem autoritären Elitenkritik“, die den Verantwortlichen „Verrat“ am eigenen Volk vorwirft, das durch die Flüchtenden bedroht sei, – werden vorrangig von den Oppositionsparteien am Rand des Spektrums (Linke, AfD) vorgebracht. Es sind auch diese Parteien, die vergleichsweise häufiger wertebezogene Frames im Rahmen ihrer öffentlichen Positionierung nutzen (vgl. die Fallzahlen zum Vorkommen von Frames im Online-Anhang). Dies schlägt sich in dem relativen Gewicht von Top-Deutungen über das gesamte Parteiensample hinweg nieder. An fünfter Stelle des Gesamtrankings findet sich der Frame, der die Sorge vor einem Rechtsruck der Gesellschaft („Pol. Kultur nach rechts“) ausdrückt.
- *CDU*: Ein deutlicher Fokus liegt auf einem Deutungsmuster, das die Forderung nach einer europäischen Lösung der Migrationskrise hervorhebt („Internationale Kooperation: Pro“). Damit sind auch sonstige öffentliche Sprecher der Partei auf einer Linie mit ihrer Vorsitzenden Angela Merkel, die sich vor allem mit diesem Frame im Jahr 2018 öffentlich positioniert hat.
- *CSU*: Auch hier ist der Frame „Internationale Kooperation: Pro“ prominent, aber es zeigt sich keine so deutliche Fokussierung wie bei der Schwesterpartei CDU. Auch die Bewertung des Frames ist hier nicht so eindeutig, an vierter Stelle der Rangordnung findet sich die ambivalente Beurteilung dieses Frames. Unter den Top-Deutungen kommen drei abgrenzende Deutungsmuster vor, die zusammengenommen ca. 40 % aller Deutungen der CSU ausmachen. Die skeptische Haltung der CSU gegenüber der Migration wird begründet mit der Sorge um die Effektivität und Handlungsfähigkeit von Regierung und Verwaltung („Effektivität des Regierens: Pro“), mit der Höherrangigkeit des sozialen Zusammenhalts der Gesellschaft, der durch weitere Migration strapaziert werde („Utilitarismus: Pro“), sowie der Position, dass Integration nur durch Assimilation der Fremden herzustellen sei („Assimilation: Pro“). Auffällig ist in diesem Zusammenhang, dass die Befürwortung einer utilitaristischen Argumentation („Utilitarismus: Pro“) sich ansonsten nur bei der CDU und der FDP wiederfindet und bei der CSU am häufigsten vorkommt.
- *SPD*: Die SPD nimmt mit Blick auf ihr Deutungsprofil eine Position ein, die vor allem internationale Kooperationen in der Migrationspolitik befürwortet („Internationale Kooperation: Pro“ sowie „Nationalstaatliche Souveränität: Con“). In dieser Hinsicht steht sie den anderen beiden Parteien innerhalb der Regierungskoalition und dem Fokus des Gesamtdiskurses nahe. Darüber hinaus problematisieren Sprecher der SPD die autoritär-abgrenzenden Tendenzen innerhalb der deutschen Gesellschaft („Pol. Kultur nach rechts: Pro“). Diesbezüglich ähnelt sie dem integrativen Profil der Grünen und der Linken.
- *GRÜNE*: Die Top-Deutungen der Grünen verdeutlichen ihren Charakter als migrationsfreundliche und kosmopolitisch ausgerichtete Partei. Am häufigsten positionieren sich die Grünen im öffentlichen Diskurs mit einer linken Elitenkritik, d. h. dem Vorwurf gegenüber der Regierung, zu wenig für die Versorgung von Migrant*innen und ihre Integration in die Gesellschaft zu tun. Die Kritik wird erweitert um die Sorge vor einem Rechtsruck der Gesellschaft und eine um sich greifende Fremdenfeindlichkeit („Pol. Kultur nach rechts: Pro“). Demgegenüber stehen die Grünen für die humanitäre Pflicht zur Hilfe für Flüchtlinge ein („Humanitarismus: Pro“). An nachgeordneter Stelle fällt ein Frame auf, den neben den Grünen vor allem die CDU in den Diskurs einspeist („Internationale Kooperation: Pro“). Im Untersuchungszeitraum spielen auf medialer und politischer Seite jene Deutungsmuster, die sich auf die ökonomische Konfliktdimension des Cleavages Integration versus Abgrenzung beziehen, nur eine randständige Rolle. Der Frame „Ökonomischer Nutzen: Pro“, der für die Zuwanderung mit dem ökonomischen Nutzen der aufnehmenden Gesellschaft wirbt, wird allerdings von den Grünen (und der CDU) verwendet und rangiert an letzter Stelle der fünf Top-Deutungen.
- *FDP*: Auch die FDP positioniert sich elitenkritisch, allerdings wird im Vergleich zu den Grünen die richtungspolitisch entgegengesetzte Position eingenommen. Neben der AfD findet sich vor allem bei der FDP die moderat autoritäre Elitenkritik, die den Regierenden Versagen bei der Reglementierung und Beschränkung der Zuwanderung vorwirft. Relativ häufig unterstützt die FDP auch die Forderung nach internationalen Lösungen in der Migrationspolitik („Internationale Kooperation: Pro“).
- *LINKE*: Das Deutungsprofil der Linken ähnelt in der Kritik am mangelnden Engagement für die Integration, an der Fremdenfeindlichkeit und im Bekenntnis zum Humanismus dem der Grünen, die Befürwortung des Frames „Linke Elitenkritik“ nimmt hier jedoch ein noch stärkeres Gewicht ein.
- *AfD*: Der Blick auf die Top-Deutungen der AfD verdeutlicht ihren Charakter als migrations- und elitenfeindliche Partei. Extrem autoritär-abgrenzende Frames wie „Wohlfahrtschauvinismus: Pro“, also der Vorwurf, die Aufnahme von Flüchtlingen entziehe den Einheimischen soziale Hilfen, auf die sie ein Recht hätten, oder die extreme Variante der Elitenkritik, die den Regierenden nicht nur Versagen bei der Begrenzung der Migration vorwirft, sondern gar eine feindselige Haltung gegenüber dem eigenen Volk, werden ausschließlich von der AfD vorgebracht. Die AfD positioniert sich ferner nicht nur in kultureller Hinsicht kritisch gegenüber Migrationsbewegungen. Der Frame „Kosten/Belastung Sozialstaat: Pro“, der auf die ökonomische Dimension des Integration-Abgrenzung-Cleavages referiert, zählt ebenfalls zu den Top-Frames der Partei.

### Deutungsprofile der Medien

Mithilfe der Deutungsprofile beschreiben wir, wie eng oder breit das inhaltliche Profil ist, das die Medien dem Thema geben (Tab. 4 im Online-Anhang).

- *FAZ*: Die häufigsten Deutungsmuster innerhalb der FAZ umfassen sowohl abgrenzende als auch integrativ ausgerichtete Frames. Es ist ein breites Spektrum unterschiedlicher Deutungen zu erkennen. Innerhalb dieser Auswahl von Frames nimmt eine Deutung eine hervorgehobene Stellung ein, die auch auf das gesamte Mediensample bezogen von höchster Prominenz ist („Internationale Kooperation: Pro“).
- *SZ*: Die SZ kennzeichnet bezogen auf alle Deutungsmuster eine mittlere argumentative Breite. Der Blick auf die Top-5-Deutungen zeigt dabei aber eine Akzentuierung von integrationsbefürwortenden Deutungsmustern (die ersten vier Ränge zusammengenommen 42 %).
- *ZEIT* und *SPIEGEL*: Ähnlich der FAZ wird in der Wochenzeitung ZEIT und dem Nachrichtenmagazin SPIEGEL insgesamt und bezogen auf die Top-5-Deutungsmuster eine argumentative Breite und richtungspolitische Ausgewogenheit deutlich. Das Repertoire der widerstreitenden Perspektiven auf das Thema wird breit vorgestellt.
- *BILD*: Mit Blick auf die Top-Deutungen innerhalb der Boulevardzeitung BILD wird eine einseitig verengte Berichterstattung in Kombination mit klar abgrenzender Tendenz deutlich. Die beiden abgrenzenden Frames „Moderat autoritäre Elitenkritik: Pro“ sowie „Innere Sicherheit: Law-and-Order: Pro“ sind dominant (mehr als die Hälfte aller Frames innerhalb des Mediums). Demnach beherrschen der Vorwurf an die Regierenden, sie versagten bei der Begrenzung der Migration, und die Betonung von Problemen bei Sicherheit und Ordnung, die aus der Migration erwüchsen, das Porträt, das die Zeitung vom Thema der Flucht- und Asylmigration zeichnet.
- *Rheinische Post*: Auch in der RP findet sich unter den Top-Frames eine argumentative Breite. Dennoch sticht die RP im Vergleich zu allen anderen Medien durch die Akzentuierung einer einzigen Deutung heraus („Internationale Kooperation“ mit 26,1 %).
- *taz* und *junge Welt*: Die Deutungsprofile der beiden „alternativen Medien“ am linken Rand des publizistischen Spektrums ähneln sich und zeigen ein deutlich integratives Profil. Besonders häufig, mit einem Anteil von circa 50 %, kommen drei Frames vor („Humanitarismus: Pro“, „Linke Elitenkritik: Pro“ und „Pol. Kultur nach rechts: Pro“).
- *Junge Freiheit*: Erwartungsgemäß stellt die Junge Freiheit den Gegenpol zur taz und zur jungen Welt dar. Die Top-Deutungen enthalten ausschließlich abgrenzende bis extrem autoritäre Frames. Der Blick auf die Rangordnung der am häufigsten vermittelten Frames korrespondiert mit der Einordnung der Zeitung als Medium der intellektuellen Neuen Rechten (u. a. Botsch 2017).

### Rangkorrelationen: Die Akzentuierung von Frames im Vergleich

Anhand der Rangkorrelationsanalyse untersuchen wir, welche Medien in der Auswahl und Akzentuierung von Frames der Argumentationsweise einzelner Parteien gleichen, d. h. deren inhaltliche Konturierung des Themas transportieren. Zudem ist von Interesse, welche Medien in der Gewichtung von Problemdeutungen eher dem Gesamtrepertoire aller Parteien ähneln.

Fasst man die Deutungen aller Parteien zusammen und bringt das Gesamtrepertoire der Partei-Frames nach der Häufigkeit der verwendeten Deutungen in eine Rangordnung, dann weisen unterschiedliche Typen von Medienangeboten eine ähnliche Rangreihe auf (Tab. 2): alternative Medien (junge Welt, Junge Freiheit), Qualitätsmedien (SZ, faz.net, sueddeutsche.de), Regionalzeitung (Rheinische Post) sowie der öffentliche-rechtliche Rundfunk (Tagesschau). Dementsprechend finden wir zudem eine relativ starke Korrelation zwischen der Rangordnung von Frames innerhalb des gesamten Parteienspektrums einerseits und dem gesamten Mediensample andererseits. Daraus ließe sich ableiten, dass zwischen Medien- und Parteiendiskurs – ungeachtet der unterschiedlichen prozentualen Gewichtungen (siehe die weiteren Analysen) – eine in moderatem Umfang gleichartige Auswahl und Priorisierung von Problemdeutungen vorliegt. Medien und Parteien fokussieren auf das Ganze gesehen im Untersuchungszeitraum ähnliche Frames. Zwei Frames haben hegemonialen Charakter („Humanitarismus“ und „Internationale Kooperation“).

**Table 2:** 

**Partei/Medium**	**BILD**	**junge Welt**	**ZEIT**	**FAZ**	**SZ**	**taz**	**Junge Freiheit**	**RP**	**SPIEGEL**	
SPD	0,049	0,363	0,179	0,342	0,475^*^	0,613^**^	−0,189	0,633^**^	0,343	
CDU	0,069	0,008	0,168	0,314	0,122	0,280	−0,185	0,335	0,199	
CSU	0,298	−0,136	0,319	0,171	−0,007	−0,074	0,236	0,067	0,191	
GRÜNE	−0,025	0,527^**^	0,174	0,295	0,547^**^	0,674^**^	−0,161	0,558^**^	0,334	
FDP	0,384	0,040	0,417^*^	0,487^*^	0,230	0,251	0,205	0,388^*^	0,302	
LINKE	−0,101	0,583^**^	0,298	0,163	0,458^*^	0,532^**^	0,018	0,464^*^	0,350	
AfD	0,412^*^	−0,022	0,070	0,105	−0,049	−0,266	0,520^**^	−0,138	0,030	
*Parteien (gesamt)*	0,444^*^	0,633^**^	*0,296*	0,434^*^	0,601^**^	0,441^*^	0,492^**^	0,544^**^	0,444^*^	
**Partei/Medium**	**Tagesschau**	**RTL aktuell**	**WDR aktuell**	**bild.de**	**spiegel.de**	**t‑online.de**	**faz.net**	**tagesschau.de**	**sueddeutsche.de**	**Medien (gesamt)**
SPD	0,481^*^	0,502^*^	0,569^**^	0,540^**^	0,540^**^	0,668^**^	0,353	0,573^**^	0,618^**^	0,557^**^
CDU	0,000	0,252	0,140	0,304	0,284	0,303	0,015	0,240	0,308	0,209
CSU	−0,008	0,188	0,008	0,173	0,126	0,074	−0,007	0,014	0,107	0,090
GRÜNE	0,500^*^	0,402^*^	0,563^**^	0,465^*^	0,544^**^	0,577^**^	0,369	0,553^**^	0,545^**^	0,546^**^
FDP	0,086	0,293	0,227	0,270	0,413^*^	0,353	0,016	0,232	0,328	0,341
LINKE	0,349	0,236	0,444^*^	0,383	0,403^*^	0,337	0,390	0,384	0,362	0,377^*^
AfD	0,087	0,000	−0,160	−0,120	−0,116	−0,108	0,029	−0,213	−0,161	−0,075
*Parteien (gesamt)*	0,651^**^	0,448^*^	0,429^*^	0,482^**^	0,454^*^	0,427^*^	0,610^**^	0,394^*^	0,470^**^	0,550^**^

^*^Die Korrelation ist auf dem 0,05-Niveau signifikant

^**^Die Korrelation ist auf dem 0,01-Niveau signifikant

Gleichwohl lassen sich auch Akzentuierungen beobachten, mit denen einzelne Medien in die Nähe zur Problemdeutung einzelner Parteien oder politischen Lager rücken. Die Selektion und Rangordnung von Frames ähneln sich vor allem zwischen SPD und Grünen und einer Reihe von Medien (taz, Rheinische Post, WDR aktuell, spiegel.de, t‑online.de, tagesschau.de und sueddeutsche.de). Die stärksten Korrelationen finden sich diesbezüglich zwischen Grünen und der taz sowie zwischen der SPD und der reichweitenstärksten Online-Plattform t‑online.de. Ferner findet sich eine signifikante Ähnlichkeit in der Rangreihe der Deutungsmuster zwischen der Partei Die Linke und der taz sowie der jungen Welt. Ins Auge fällt darüber hinaus die Korrelation zwischen dem Lagerorgan der Neuen Rechten, der Jungen Freiheit, und der AfD. Dass sich die Akzentuierung von Frames zwischen Medium und Partei im Migrationsdiskurs stark ähnelt, erscheint plausibel.

### Kontingenzanalyse: Positionen im Konflikt zwischen Integration und Abgrenzung

Die Kreuztabelle gibt ein Bild von der Verteilung der Frames auf die Pole der Konfliktdimension Integration versus Abgrenzung. Zusammengefasst und dem integrativen Pol zugeordnet werden im Rahmen der Auswertung alle Deutungsmuster, die im Sinne von Kriesi et al. eher integrationsfreundlich sind. Umgekehrt werden alle Perspektiven, die die Aufnahme von Flüchtlingen ablehnen und die Politik dafür kritisieren, dem abgrenzenden Pol zugeordnet. Tab. 3 zeigt, wie eindeutig und einseitig die argumentative Positionierung von Parteien in dieser Konfliktdimension ist. Für die Medien wird verdeutlicht, wie nahe sie mit der prozentualen Gewichtung von Frames für je eine Seite den verschiedenen Polen im Konflikt sind.

**Table 3:** 

Medium/Partei	Integration	Abgrenzung
LINKE	98,4	1,6
GRÜNE	93,8	6,3
taz	76,9	23,1
SPD	75,9	24,1
tagesschau.de	68,6	31,4
junge Welt	65,4	34,6
spiegel.de	64,8	35,2
bild.de	64,8	35,2
Tagesschau	64,0	36,0
sueddeutsche.de	62,3	37,7
WDR aktuell	60,0	40,0
t‑online.de	59,0	41,0
CDU	58,7	41,3
SZ	57,7	42,3
*Medien (gesamt)*	57,6	42,4
ZEIT	56,3	43,8
faz.net	54,4	45,6
RP	52,9	47,1
Spiegel	45,0	55,0
RTL aktuell	44,0	56,0
*Parteien (gesamt)*	41,6	58,4
FAZ	35,7	64,3
Junge Freiheit	20,0	80,0
CSU	18,8	81,3
BILD	18,4	81,6
FDP	16,4	83,6
AfD	1,4	98,6

Parteien: χ^2^ = 507,28, df = 6, *p* < 0,001, Cramer-V = 0,86; Medien: χ^2^ = 134,44, df = 17, *p* < 0,001, Cramer-V = 0,27

In der Gesamtschau ist zu erkennen, dass das Deutungsrepertoire in den Medien verglichen mit dem Spektrum der Positionen der Parteien in Richtung des integrativen Konfliktpols verschoben ist.^7^ Eine Auswahl von Medien steht bei der Wiedergabe von Deutungsmustern der Mitte der Parteipositionierungen relativ nahe und gibt den Elitendiskurs – nach Maßgabe der von den Parteien vertretenen Deutungsmuster – dementsprechend ausgewogen wieder (Spiegel, RTL aktuell, FAZ).

Andere Medien sind vom Parteiendurchschnitt weiter entfernt und stehen mit der Tendenz der von ihnen übermittelten Frames einzelnen Parteien näher. Darin lässt sich ein Anhaltspunkt für mögliche Diskurskoalitionen mit Parteien erkennen. So steht vor allem die taz den Parteien des (moderat) linken Lagers nahe. Eine Reihe von Medien (u. a. SZ und ZEIT) entspricht der CDU-Position, d. h. mehr oder weniger ausgewogen mit leichter Tendenz zum Integrationspol. Die BILD und die Junge Freiheit finden sich in der Nähe derjenigen Parteien, die sich mit abgrenzenden Frames öffentlich positionieren. Die Bedeutung von redaktionellen Linien für eine richtungspolitisch einseitige Berichterstattung zeigt sich vor allem bei Medien an den Rändern des publizistischen Spektrums. Dies begünstigt inhaltliche Parallelstrukturen zu Parteien am Rand des politischen Spektrums. Eine besondere Rolle spielt das Boulevardblatt BILD. Das von BILD übermittelte (eingeschränkte) Repertoire an Deutungen zum Thema Fluchtmigration privilegiert klar ausgrenzende Positionen.

### RD-Index: Allianzen und Distanzen im Konflikt zwischen Integration und Abgrenzung

Als Erweiterung der zuvor referierten Kontingenzanalyse wird mithilfe des Reflective-Diversity-Indexes der Versuch unternommen, die Nähe bzw. Distanz von Medien und Parteien in einer Maßzahl zu bündeln und dabei auch den diskutierten Maßstab einer angemessenen Repräsentation der konkurrierenden Parteipositionen nach dem Prinzip der abgestuften Chancengleichheit zu berücksichtigen. Der Koeffizient gibt in einer Maßzahl zwischen null (geringe Übereinstimmung) und eins (hohe Übereinstimmung) an, wie inhaltlich nahe oder distanziert sich Parteien und Medien bei der Deutung des Konfliktthemas Flucht- und Asylmigration sind (Tab. 4).

**Table 4:** 

**Partei**	**BILD**	**junge Welt**	**ZEIT**	**FAZ**	**SZ**	**taz**	**Junge Freiheit**	**RP**	**SPIEGEL**	
CDU	0,60	*0,93*	*0,97*	0,77	*0,99*	*0,82*	0,61	*0,95*	*0,87*	
CSU	*1,00*	0,53	0,63	*0,83*	0,61	0,42	*0,99*	0,65	0,73	
SPD	0,43	*0,89*	*0,80*	0,60	*0,82*	*0,99*	0,44	0,77	0,70	
GRÜNE	0,25	0,72	0,62	0,42	0,64	*0,83*	0,26	0,60	0,52	
FDP	*0,97*	0,51	0,61	*0,81*	0,58	0,39	*0,96*	0,63	0,71	
LINKE	0,21	0,67	0,57	0,37	0,60	0,79	0,21	0,55	0,47	
AfD	*0,82*	0,36	0,46	0,66	0,43	0,24	*0,82*	0,48	0,56	
**Parteien (gesamt)**	0,75	0,79	*0,88*	*0,92*	*0,86*	0,67	0,76	*0,91*	*0,98*	
**Partei**	**Tagesschau**	**RTL aktuell**	**WDR aktuell**	**bild.de**	**spiegel.de**	**t‑online.de**	**faz.net**	**tagesschau.de**	**sueddeutsche.de**	**Medien (gesamt)**
CDU	*0,95*	*0,85*	*0,97*	*0,94*	*0,94*	*1,00*	*0,95*	*0,90*	*0,96*	*0,99*
CSU	0,55	0,75	0,57	0,54	0,54	0,60	0,65	0,50	0,56	0,61
SPD	*0,88*	0,68	*0,86*	*0,89*	*0,89*	*0,83*	0,78	*0,93*	*0,87*	*0,82*
GRÜNE	0,70	0,50	0,68	0,71	0,71	0,65	0,60	0,75	0,69	0,64
FDP	0,53	0,73	0,55	0,52	0,52	0,57	0,62	0,48	0,54	0,59
LINKE	0,65	0,45	0,63	0,66	0,66	0,61	0,56	0,70	0,64	0,59
AfD	0,38	0,58	0,40	0,37	0,37	0,42	0,47	0,33	0,39	0,44
**Parteien (gesamt)**	*0,80*	*1,00*	*0,83*	0,79	0,79	*0,85*	*0,90*	0,75	*0,81*	*0,86*

Index-Werte von über 0,8, die eine hohe Übereinstimmung von Parteien und Medien anzeigen, sind kursiv gedruckt

Eine Reihe von Medien reflektiert die Mitte der Parteipositionen auf der Konfliktdimension Integration versus Abgrenzung. Neben Spiegel und RTL aktuell haben hier auch die Rheinische Post, FAZ und ihr Online-Pendant faz.net Spitzenwerte über 0,9. Eine große Nähe der Positionierung von Parteien und Medien zeigt sich anhand dieser Auswertungsvariante für BILD-CSU-FDP, für ZEIT-CDU, SZ-CDU, taz-SPD, Junge Freiheit-CSU-FDP, WDR aktuell-CDU, t‑online.de-CDU sowie sueddeutsche.de-CDU. Hier haben die Koeffizienten nahezu den höchsten Wert 1,0. Die Auswertung des Reflective-Diversity-Indexes unterstreicht zum Teil die im Rangkorrelationstest identifizierte Nähe von einzelnen Medien und Parteien (taz und SPD). Durch die in dieser Analyse berücksichtigte, prozentuale Gewichtung von Deutungsmustern können aber auch weitere Diskurskoalitionen identifiziert werden. So ist beispielsweise die CDU mit ihrer moderat-integrativen Position in mehreren Medien perfekt repräsentiert. Vor allem Qualitätsmedien bilden das Repertoire der Deutungsmuster auf richtungspolitisch ausgewogene Weise ab. Daraus ergibt sich für diese Angebote in der Gesamttendenz der Berichterstattung eine gewisse Nähe zu moderat positionierten Parteien wie der CDU.

Bezüglich der analysierten Rundfunkangebote sowie Online-Medien ist auf eine Besonderheit hinzuweisen. Die Auswahl von Frames ist hier im Vergleich zum Print-Sektor eingeschränkt und inhaltlich relativ homogen, das Deutungsrepertoire wird in seiner Breite nicht ausgeschöpft. Diese Angebote fokussieren im Untersuchungszeitraum sehr stark auf ein zentrales, integratives Deutungsmuster („Humanitarismus“). Verrechnet mit dem vergleichsweise geringen Anteil anderer Frames ergibt sich hieraus jeweils eine richtungspolitische Position, die der Regierungspartei CDU nahe bis sehr nahe steht. Auch das im Vergleich zum Print-Pendant eher überraschende integrative Profil von bild.de lässt sich auf diese Weise erklären.^8^ Darüber hinaus zeigt auch der Blick auf den Wert für das Mediensample insgesamt eine nahezu vollständige Übereinstimmung mit der relativ moderaten Position der CDU in der Konfliktdimension Integration versus Abgrenzung.

Eine besonders große Distanz findet sich mit Koeffizienten um 0,2 zwischen der BILD und der Jungen Freiheit auf der Medienseite und Grünen sowie Linken auf der Parteienseite. Auf der richtungspolitischen Gegenseite zeigt sich hingegen eine große Distanz zwischen der taz und der AfD-Position.

## Fazit

Studien zum Medien-Parteien-Parallelismus geben darüber Auskunft, inwieweit Medien allein der Fortschreibung partikularer Weltanschauungen in politischen Lagern dienen oder durch eine umfassende und ausgewogene Wiedergabe konkurrierender Problemdeutungen ihren Nutzern Grundlagen für eine unabhängige individuelle Meinungsbildung verschaffen. Die Freiheit der Nutzer, sich gestützt auf die von den Medien bereitgestellten Informationen eine Meinung neu zu bilden, ist zugleich eine Voraussetzung für die Offenheit des Parteienwettbewerbs. Denn der lebt davon, dass Parteien mit ihren Problemdeutungen und Politikoptionen auch die erreichen können, die sie bisher noch nicht von sich überzeugt haben.

Die vorliegende Studie untersucht die Nähe von Medien zu Parteien anhand der Inhalte von Problemdeutungen. Dafür werden Frames anhand von Standardargumenten erfasst. Mithilfe des Konzepts werden problembezogene Deutungen zum Streitthema der Fluchtmigration identifiziert und anhand ihres Bezugs auf die Wertepole des grundlegenden Cleavages „integration versus demarcation“ klassifiziert. Das ermöglicht in mehrfacher Hinsicht eine weitergehende Aufklärung über die Rolle der Medien im politischen Konflikt.

Mit Blick auf das öffentlichkeitstheoretisch relevante Problem der Binnenpluralität kann nicht allein die Ein- oder Zweiseitigkeit in der Tendenz wiedergegebener Aussagen bestimmt werden. Darüber hinaus wird die Deutung des Problems zum Vorschein gebracht, die der Tendenz zugrunde liegt. Das erschließt ein tiefenscharfes Bild vom Charakter der medial in Umlauf gebrachten Problemdeutungen. So wird beispielsweise erkennbar, dass die Darstellung, die das Boulevardblatt BILD dem Phänomen der Flucht- und Asylmigration gibt, nahezu vollständig auf das Drohbild der Risiken für Recht und Ordnung sowie das damit verbundene vermeintliche Versagen der Zuständigen beschränkt ist. Mit dieser engen Fokussierung formiert das Massenblatt eine Anschauung, die der rechtspopulistischen Ideologie entspricht. BILD verschafft ihr einen Resonanzraum, der zur Verschiebung des öffentlichen Meinungsklimas zugunsten ausgrenzender Positionen und zum Aufstieg des organisierten Rechtspopulismus im Zuge der Migrationsdebatte beigetragen haben dürfte.

Der Rechtspopulismus hat in der Jungen Freiheit sein publizistisches Organ. Es zeichnet ein Droh- und Schreckbild von den Folgen der Migration und wendet diese Deutung zu einer scharfen Anklage gegen die Verantwortlichen. Die Junge Freiheit reproduziert damit die Wirklichkeitskonstruktion, die die AfD propagiert.

In den Medien taz und junge Welt beherrschen integrationsfreundliche Problemdeutungen das Bild. Ihre Berichterstattung gibt damit vor allem solchen Standardargumenten Raum, mit denen die Parteien Die Linke sowie Die Grünen Position beziehen. Eine Hervorhebung verdient, dass die junge Welt in der Rangreihe der wiedergegebenen Argumente dem Gesamtrepertoire *aller* Parteien ähnelt, was als Indiz dafür gelesen werden kann, dass über die Problemdeutungen des linken Lagers hinaus auch Argumente der anderen Parteien aufgegriffen und behandelt werden.

Die Analyse der Berichterstattung über das Konfliktthema der Asyl- und Fluchtmigration bestätigt schließlich erneut, dass die überregionalen „Qualitätsmedien“ ihren Titel weiterhin verdienen. FAZ, Spiegel, ZEIT und SZ bieten vor allem in ihrer klassischen gedruckten Ausgabe eine Problembeschreibung von großer argumentativer Breite und positionsbezogener Ausgewogenheit.

Mit der Erfassung von problembezogenen Deutungen lässt sich in diesem Sinn inhaltlich ermessen, welche Medien ein *Forum* für die offene Auseinandersetzung zwischen der ganzen Bandbreite politischer Positionen schaffen und welche als *Organ* einer partikularen Weltanschauung fungieren.

Mit Blick auf den Medien-Parteien-Parallelismus lässt sich nicht nur beschreiben, in welchen Medien welche Partei die Chance hat, als Sprecher in Erscheinung zu treten. Darüber hinaus lässt sich rekonstruieren, welchen *inhaltlichen Positionen* von Parteien Medien einen gesellschaftlichen Resonanzraum verschaffen, m. a. W. welches Medium welcher Partei die Möglichkeit gibt, dass ihre Politikangebote vor die Augen des von den Medien geschaffenen Publikums treten können. Dadurch wird beschreibbar, was Medien für einen lebendigen *politischen Pluralismus* leisten. Denn anders und gesellschaftlich viel stärker als Social-Media-Kanäle haben Medien das Potenzial, das Spektrum politischer Positionen lagerübergreifend wahrnehmbar und diskutierbar zu machen. Unsere Analyse für den Fall eines herausragenden Konfliktthemas ergibt: Es sind vor allem die den klassischen journalistischen Standards verpflichteten überregionalen Qualitätsmedien, die im öffentlichen Diskurs die kommunikativen Grundlagen für politischen Pluralismus lebendig halten. Das gilt schon für ihre Online-Ableger nur noch mit gewissen Einschränkungen. Die sind bei den Medien für ein linkes politisches Publikum deutlicher ausgeprägt. Junge Freiheit und das Boulevardblatt BILD lassen nur noch eine rechte bzw. rechtspopulistische Weltanschauung vorkommen.

Der analytische Nutzen einer Untersuchung des politischen Parallelismus mit problembezogenen Deutungen ist in der vorliegenden Studie allerdings noch in drei Hinsichten nur begrenzt ausgeschöpft. Unsere Studie beschreibt die *Parallelität* der Deutungsprofile von Medien und Parteien. In einem weiteren Schritt könnte im Sinne einer Input-Output-Analyse ermittelt werden, welche von den Parteien selbst artikulierten Positionen von Medien übermittelt und dabei den Sprechern klar zugeordnet werden. Dann ließe sich prüfen, inwieweit Medien ein übereinstimmendes Bild von den inhaltlichen Positionen der Parteien zeichnen oder ob sie ihren Nutzern ein je unterschiedlich akzentuiertes *Porträt* der Parteiprofile geben. Dafür reicht in der vorliegenden Studie die Anzahl der erfassten Beiträge, in denen Parteiakteure als Sprecher vorkommen, nicht aus.

Um das Bild vom politischen Parallelismus zu komplettieren, lohnt es sich, neben Medien und Politik auch die dritte zentrale Akteursgruppe politischer Kommunikation, d. h. das Publikum bzw. die Bürgerschaft in den Blick zu nehmen (McNair 2003; Pfetsch 2014). Anhand von Befragungsstudien könnten die Problemwahrnehmungen, politischen Einstellungsmuster und Kommunikationspraktiken verschiedener Bevölkerungssegmente identifiziert und mit den Befunden zum Parallelismus von Medien und Parteien in Beziehung gesetzt werden. Es ließe sich mit einer solchen Verknüpfung von Inhalts- und Nutzungsanalyse ermitteln, welche Bevölkerungssegmente von medialen Angeboten erreicht werden, die politische Konflikte auf plurale Weise vermitteln und welche sich nur aus inhaltlich einseitigen Lagerorganen ihr Weltbild formen. Auf diese Weise können weitergehende Fragestellungen bearbeitet werden, die sich mit gesellschaftlichen Herausforderungen wie etwa einem geringen medialen Repräsentationsgefühl oder politisch-medialen Polarisierungstendenzen in einzelnen Bevölkerungssegmenten auseinandersetzen (Bennett und Iyengar 2008).

Unsere Diagnose von Nähe und Distanz von Medien zu Parteien beschränkt sich auf ein einziges Thema von hoher Relevanz in einem begrenzten Untersuchungszeitraum. Es ist daher nicht klar, inwieweit sich bei anderen Streitthemen gleichartige Relationen von Nähe und Distanz zwischen Medien und Parteien auffinden lassen. Dazu müsste der Bezug zu den Wertepolen der politischen Cleavages *themenübergreifend* erfasst werden.^9^ Dann ließe sich präziser charakterisieren, welche Partei mit welchen Themen und welchen normativen Grundpositionen in welchen Medien wahrnehmbar wird. Auf dieser Grundlage könnte die politikwissenschaftliche Klassifikation von Parteipositionen, wie sie im MARPOR-Projekt (Wissenschaftliches Zentrum Berlin für Sozialforschung 2020) vorgenommen wird, mit den Einsichten darüber verknüpft werden, was davon Medien gesellschaftlich sichtbar machen. Eine solche Verknüpfung politik- und kommunikationswissenschaftlicher Ansätze verspricht weiterführende Einsichten in das Funktionieren der politischen Meinungs- und Willensbildung in der Gesellschaft.

## Supplementary Information






## Footnotes

1 In diesem Text verwenden wir die Begriffe Problemdeutung, Deutungsmuster und Frame synonym.

2 Vor unseren Analysen haben wir den Datensatz gewichtet, um die ursprünglichen Anteile der Medienangebote an der Grundgesamtheit wiederherzustellen. Eine detaillierte Beschreibung der Stichprobenbildung samt Verweisen auf weiterführende Literatur findet sich in Kösters (2020, S. 129–133).

3 Die Labels der Frames in Tab. 1 geben Hinweise auf den inhaltlichen Gehalt der jeweiligen Deutungsmuster. Eine detaillierte Übersicht zu den Inhalten der Frames kann im Online-Anhang samt der Angabe von verwendeten Quellen eingesehen werden.

4 Theoretisch ist eine zusätzliche Zuordnung ausgewählter Frames zur ökonomischen Dimension möglich. Im Untersuchungszeitraum fanden sich jedoch fast nur solche Frames, die sich inhaltlich auf die kulturelle Dimension beziehen.

5 Die Deutungsprofile aller anderen untersuchten Medien können in Tab. 4 im Online-Anhang eingesehen werden.

6 Während eines Teils des Untersuchungszeitraumes stand die Kontroverse zwischen Bundeskanzlerin Merkel und dem Innenminister Seehofer über ein europäisches versus nationales Management der Migrationskrise im Fokus der Berichterstattung. Wir interessieren uns für das Verhältnis von integrativen versus abgrenzenden Standpunkten *jenseits* dieser singulären Debatte. Daher schließen wir die beiden Frames „Internationale Kooperation“ und „Nationale Souveränität“ bei der Berechnung des relativen Gewichts der Konfliktpole aus. Darüber hinaus ist der Frame der „Internationalen Kooperation“ nicht zwingend als integrativ einzuordnen. So wäre beispielsweise ein Verweis auf mehr Zusammenarbeit zwischen europäischen Ländern nicht mehr integrativ, wenn er mit der Forderung nach einer stärkeren Abgrenzung nach außen verbunden würde (vgl. die Kritik an einer „Festung Europa“). Der Vollständigkeit halber ergänzen wir im Online-Anhang eine Kontingenztabelle (Tab. 4) zur richtungspolitischen Verortung von Medien und Parteien *inklusive* der beiden genannten Deutungsmuster.

7 Dabei ist zu beachten, dass die besonders hohe Zahl von Frames der AfD den Durchschnitt der Parteien hin zum Abgrenzungspol bewegt. Eine Analyse, die die relative politische Bedeutung einzelner Parteien in Rechnung stellt, erfolgt im nächsten Schritt.

8 Siehe dazu Tab. 4 im Online-Anhang.

9 Eine solche Klassifikation von Akteurspositionen auf der abstrahierenden Ebene der Wertepositionen wird in einer weiteren Studie des Projekts „Media Performance and Democracy“ durchgeführt. Ergebnisse werden an anderer Stelle veröffentlicht. Siehe zur Projektbeschreibung https://mediaperformance.uni-mainz.de/.
