# Supplementary material for: Discursive Alliances in the Debate on Migration? Political Parallelism Between Media and Parties in the Framing of the 2018 Debate on Refugee and Asylum Migration
Source: Polit Vierteljahresschr. 2021 Jul 7;62(3):461–87. [Article in German] doi: 10.1007/s11615-021-00324-z (PMC8550640; doi:10.1007/s11615-021-00324-z)
Supplement: Supplementary file 1 [file 11615_2021_324_MOESM1_ESM.pdf]

Diskursallianzen in der Migrationsdebatte?

Politischer Parallelismus zwischen Medien und Parteien im Framing der Flucht- und Asylmigration im Jahr 2018

Ergänzende Tabellen zum Beitrag

Tabelle 1: Übersicht Parteiensample

Partei	Presse-mitteilungen	Bundestags-reden	Positions-papiere	Dokumente gesamt	Standardargumente gesamt
SPD	38	58	3	99	64
CDU	129	67	2	198	126
CSU	145	31	4	180	81
Grüne	88	37	3	128	121
FDP	44	31	4	79	90
Linke	82	37	1	120	133
AfD	173	81	1	255	344
**Gesamt**	699	342	18	1059	959

Tabelle 2: Übersicht Mediensample

Medium	Beiträge gesamt	Standardargumente gesamt
FAZ	200	341
SZ	208	333
ZEIT	61	171
SPIEGEL	76	210
BILD	136	128
RP	171	202
taz	172	275
junge Welt	152	200
Junge Freiheit	126	253
Tagesschau	129	110
RTL aktuell	164	153
WDR aktuell	99	82
t-online.de	93	95
bild.de	84	187
spiegel.de	86	71
sueddeutsche.de	80	139
faz.net	81	92
tagesschau.de	79	117
**Gesamt**	2197	3159

Tabelle 3: Deutungsprofile der Parteien (absolute Häufigkeiten, Rang in Klammern)

**Deutungsmuster und Valenz**	**SPD**	**CDU**	**CSU**	**Grüne**	**FDP**	**Linke**	**AfD**	**Gesamt**
Internationale Kooperation: Pro	25 (1)	57 (1)	15 (1)	13 (3)	20 (2)	4 (4)	7 (10)	141
Linke Elitenkritik: Pro	2 (6)	-	1 (10)	50 (1)	-	82 (1)	2 (12)	137
Moderat autoritäre Elitenkritik: Pro	6 (4)	2 (7)	3 (8)	5 (6)	37 (1)	-	83 (1)	136
Extrem autoritäre Elitenkritik: Pro	-	-	2 (9)	-	-	-	53 (2)	55
Pol. Kultur nach rechts: Pro	10 (2)	5 (4)	-	17 (2)	1 (6)	19 (2)	-	52
Innere Sicherheit: Law-and-Order: Pro	-	1 (8)	1 (10)	-	-	-	44 (3)	46
Humanitarismus: Pro	8 (3)	5 (4)	1 (10)	12 (4)	2 (5)	18 (3)	-	46
Effektivität des Regierens: Pro	1 (7)	6 (3)	11 (2)	1 (8)	5 (3)	1 (5)	11 (7)	36
Kosten und Belastung Sozialstaat: Pro	-	-	3 (8)	-	1 (6)	-	30 (4)	34
Pol. Kultur nach links: Pro	-	1 (8)	4 (7)	-	1 (6)	1 (5)	25 (6)	32
Wohlfahrtschauvinismus: Pro	-	-	-	-	-	-	29 (5)	29
Utilitarismus: Pro	-	4 (5)	9 (3)	-	4 (4)	-	3 (11)	20
Ökonomischer Nutzen: Pro	3 (5)	7 (2)	-	7 (5)	1 (6)	1 (5)	-	19
Internationale Kooperation: Ambiv.	1 (7)	6 (3)	8 (4)	-	1 (6)	-	2 (12)	18
Assimilation: Pro	-	1 (8)	6 (5)	-	-	-	9 (9)	16
Republikanismus: Pro	1 (7)	5 (4)	2 (9)	1 (8)	4 (4)	-	2 (12)	15
Nationalstaatliche Souveränität: Con	6 (4)	3 (6)	-	3 (7)	-	-	-	12
Ökonomischer Nutzen: Con	-	1 (8)	-	-	-	-	10 (8)	11
**Standardargumente pro Partei (n)**	64	126	81	121	90	133	344	959

Tabelle 4: Deutungsprofile der untersuchten Medienangebote (in Prozent, Rang in Klammern)

**Deutungsmuster und Valenz**	**BILD**	**Junge Welt**	**ZEIT**	**FAZ**	**SZ**	**taz**	**Junge Freiheit**	**RP**	**Spiegel**
Internationale Kooperation: Pro	4,3 (4)	6,6 (4)	4,7 (3)	14,4 (1)	12,5 (1)	8,9 (4)	3,2 (6)	26,1 (1)	5,5 (4)
Humanitarismus: Pro	6,5 (3)	19,8 (1)	11,6 (1)	5,2 (4)	12,5 (1)	18,4 (1)	5,4 (5)	6,8 (3)	12,7 (1)
Linke Elitenkritik: Pro	-	16,5 (2)	2,3 (4)	1,5 (10)	10,1 (2)	12,8 (2)	2,2 (7)	4,5 (4)	3,6 (5)
Moderat autoritäre Elitenkritik: Pro	28,3 (1)	2,2 (6)	4,7 (3)	5,2 (4)	5,3 (5)	3,4 (5)	6,5 (4)	8,0 (2)	7,3 (3)
Effektivität des Regierens: Pro	6,5 (3)	2,2 (6)	7,0 (2)	8,2 (2)	6,3 (4)	2,2 (6)	6,5 (4)	6,8 (3)	9,1 (2)
Pol. Kultur nach rechts: Pro	2,2 (5)	13,2 (3)	4,7 (3)	3,6 (6)	6,7 (3)	10,1 (3)	2,2 (7)	8,0 (2)	7,3 (3)
Innere Sicherheit: Law-and-Order: Pro	21,7 (2)	5,5 (5)	2,3 (4)	6,2 (3)	2,9 (7)	2,2 (6)	11,8 (2)	2,3 (5)	3,6 (5)
Republikanismus: Pro	2,2 (5)	-	4,7 (3)	4,1 (5)	1,4 (9)	1,7 (7)	2,2 (7)	-	1,8 (6)
Nationalstaatliche Souveränität: Con	-	-	--	2,1 (9)	1,0 (10)	1,7 (7)	-	1,1 (6)	1,8 (6)
Extrem autoritäre Elitenkritik: Pro	4,3 (4)	2,2 (6)	2,3 (4)	3,6 (6)	2,4 (8)	0,6 (9)	12,9 (1)	-	3,6 (5)
Internationale Kooperation: Ambiv.	2,2 (5)	-	2,3 (4)	1,5 (10)	0,5 (11)	1,7 (7)	-	2,3 (5)	1,8 (6)
Wohlfahrtschauvinismus: Pro	2,2 (5)	2,2 (6)	4,7 (3)	2,6 (8)	3,4 (6)	2,2 (6)	3,2 (6)	2,3 (5)	1,8 (6)
Utilitarismus: Pro	2,2 (5)	-	2,3 (4)	2,6 (8)	1,0 (10)	1,1 (8)	3,2 (6)	1,1 (6)	3,6 (5)
Pol. Kultur nach links: Pro	-	-	4,7 (3)	1,5 (10)	0,5 (11)	0,6 (9)	7,5 (3)	-	1,8 (6)
Kosten und Belastung Sozialstaat: Pro	4,3 (4)	1,1 (7)	2,3 (4)	3,1 (7)	1,0 (10)	0,6 (9)	3,2 (6)	1,1 (6)	-
Ökonomischer Nutzen: Pro	-	2,2 (6)		3,1 (7)	1,0 (10)	1,7 (7)	2,2 (7)	2,3 (5)	-
Assimilation: Pro	2,2 (5)	1,1 (7)	4,7 (3)	1,5 (10)	1,0 (10)	0,6 (9)	2,2 (7)	-	1,8 (6)
Ökonomischer Nutzen: Con	-	-	-	-	-	0,6 (9)	-	-	1,8 (6)
**Standardargumente pro Medium (n)**	128	200	171	341	333	275	253	202	210

Fortsetzung Tabelle 4

Deutungsmuster und Valenz	Tagesschau	RTL aktuell	WDR aktuell	bild.de	spiegel.de	t-online.de	faz.net	tagesschau.de	sueddeutsche.de	Gesamt
Internationale Kooperation: Pro	22,2 (1)	17,9 (1)	19,2 (1)	22,5 (1)	16,1 (2)	13,8 (1)	9,8 (2)	22,8 (1)	15,7 (1)	14,8
Humanitarismus: Pro	16,7 (2)	10,3 (2)	15,4 (2)	22,0 (2)	22,0 (1)	13,8 (1)	18,4 (1)	15,8 (2)	11,1 (2)	14,8
Linke Elitenkritik: Pro	5,6 (3)	2,6 (4)	7,7 (3)	9,8 (3)	6,0 (5)	6,3 (5)	6,3 (3)	7,6 (3)	9,1 (4)	7,3
Moderat autoritäre Elitenkritik: Pro	5,6 (3)	7,7 (3)	3,8 (4)	4,4 (6)	7,1 (4)	12,7 (2)	4,0 (4)	2,7 (7)	6,6 (5)	7,1
Effektivität des Regierens: Pro	2,8 (4)	7,7 (3)	7,7 (3)	4,9 (5)	6,0 (5)	8,5 (3)	4,0 (4)	4,9 (4)	6,6 (5)	6,2
Pol. Kultur nach rechts: Pro	2,8 (4)	7,7 (3)	3,8 (4)	2,7 (7)	6,0 (5)	7,3 (4)	2,3 (6)	4,3 (5)	10,6 (3)	6,0
Innere Sicherheit: Law-and-Order: Pro	2,8 (4)	10,3 (2)	-	2,2 (8)	1,2 (7)	2,1 (8)	6,3 (3)	1,1 (9)	4,5 (6)	3,6
Republikanismus: Pro	-	2,6 (4)	-	1,0 (9)	8,9 (3)	5,2 (6)	-	2,7 (7)	1,5 (9)	2,9
Nationalstaatliche Souveränität: Con	2,8 (4)	2,6 (4)	7,7 (3)	2,7 (7)	3,0 (6)	4,2 (7)	3,4 (5)	4,3 (5)	3,0 (8)	2,7
Extrem autoritäre Elitenkritik: Pro	5,6 (3)	2,6 (4)	-	2,2 (8)	3,0 (6)	2,1 (8)	4,0 (4)	2,7 (7)	1,5 (9)	2,7
Internationale Kooperation: Ambiv.	-	2,6 (4)	-	6,4 (4)	-	2,1 (8)	1,1	1,6 (8)	3,5 (7)	2,2
Wohlfahrtschauvinismus: Pro	2,8 (4)	-	3,8 (4)	1,0 (9)	-	1,0 (9)	4,0 (4)	3,3 (6)	-	1,8
Utilitarismus: Pro	-	-	-	1,0 (9)	1,2 (7)	-	1,1 (7)	1,6 (8)	1,5 (9)	1,1
Pol. Kultur nach links: Pro	-	-	-	1,0 (9)	1,2 (7)	-	1,1 (7)	-	-	0,8
Kosten und Belastung Sozialstaat: Pro	-	2,6 (4)	3,8 (4)	-	-	-	1,1 (7)	-	0,5 (10)	0,8
Ökonomischer Nutzen: Pro	-	-	-	-	-	-	2,3 (6)	-	-	0,7
Assimilation: Pro	-	2,6 (4)	-	-	-	-	1,1 (7)	-	-	0,6
Ökonomischer Nutzen: Con	-	-	-	-	-	-	-	-	0,5 (10)	0,1
**Standardargumente pro Medium (n)**	110	153	82	187	71	95	92	117	139	3159

[a]Wir konzentrieren uns in unseren Analysen auf die 18 innerhalb des gesamten Parteiensamples häufigsten Deutungsmuster. Deutungsmuster, die seitens der Medien relativ häufig vermittelt werden, von den Parteien aber selten bis gar nicht, wurden in diese Tabelle nicht übernommen.

Tabelle 5: Integrationsfreundliche und abgrenzende Deutungsmuster von Parteien und Medienangeboten inklusive der Frames „Nationale Souveränität" und „internationale Kooperation" (in Prozent)

**Medium / Partei**	**Integration**	**Abgrenzung**
LINKE	98,5	1,5
GRÜNE	94,6	5,4
SPD	88,3	11,7
CDU	81,3	18,7
taz	75,3	24,7
tagesschau.de	74,8	25,2
bild.de	74,4	25,6
Tagesschau	71,4	28,6
spiegel.de	70,6	29,4
junge Welt	66,7	33,3
sueddeutsche.de	66,3	33,7
RP	65,4	34,6
WDR aktuell	65,2	34,8
**Medien(gesamt)**	63,3	36,7
t-online.de	63,1	36,9
SZ	63,1	36,9
faz.net	57,5	42,5
ZEIT	57,1	42,9
RTL aktuell	52,9	47,1
**Parteien (gesamt)**	51,5	48,5
Spiegel	48,9	51,1
FAZ	46,8	53,2
FDP	37,0	63,0
CSU	36,4	63,6
BILD	23,3	76,7
Junge Freiheit	21,6	78,4
AfD	3,7	96,3

[b] Parteien: $\chi^2 = 553{,}36$, df = 6, $p < 0{,}001$, Cramer-V = 0,81; Medien: $\chi^2 = 165{,}99$, df = 17, $p < 0{,}001$, Cramer-V = 0,26
