# Supplementary material for: Discursive Alliances in the Debate on Migration? Political Parallelism Between Media and Parties in the Framing of the 2018 Debate on Refugee and Asylum Migration
Source: Polit Vierteljahresschr. 2021 Jul 7;62(3):461–87. [Article in German] doi: 10.1007/s11615-021-00324-z (PMC8550640; doi:10.1007/s11615-021-00324-z)
Supplement: Supplementary file 2 [file 11615_2021_324_MOESM2_ESM.pdf]

Diskursallianzen in der Migrationsdebatte?

Politischer Parallelismus zwischen Medien und Parteien im Framing der Flucht- und Asylmigration im Jahr 2018

Codebuch

Anmerkung: Der oben genannte Beitrag ist im Rahmen des Forschungsprojektes „Media Performance & Democracy“ entstanden. Dieses Codebuch stellt einen Auszug aus dem umfangreicheren Gesamt-Codebuch des Projektes dar. An dieser Stelle werden nur jene Kategorien und Codieranweisungen beschrieben, die für den dem Beitrag zugrundeliegenden Datensatz von Relevanz sind. Das Codebuch bezieht sich auf die Inhaltsanalyse der Mediendokumente. Für die Analyse der Parteiendokumente kann es – unter Ausschluss der medienspezifischen Hinweise – ebenfalls eingesetzt werden.

Inhaltsverzeichnis

Deutungsmuster: Standardargumente (STA) 3

Liste möglicher Deutungsmuster und dazugehörige Codes 4

Deutungsmuster: Valenz (STA_VAL) 29

Sprecher und Akteure – Vorbemerkung und übergeordnete Hinweise 31

Einzelpersonen und unabgeschlossene Personengruppen (SPRECH_EIN) 33

Name des Sprechers/Akteurs (SPRECH_NAM) 34

Zuordnung von Sprechern/Akteuren zu Institutionen und Organisationen (SPRECH) 35

Parteizugehörigkeit von Sprechern/Akteuren (SPRECH_PAR) 45

Literatur 47

Deutungsmuster: Standardargumente (STA)

Die im Text identifizierten wertebezogenen Aussagen sollen mithilfe dieser Kategorie einer Ausprägung der Liste an Deutungsmustern und den dazugehörigen Codes zugeordnet werden. Es gibt prinzipiell *keine zahlenmäßige Begrenzung*, es sollen pro Artikel/Beitrag alle von einem Sprecher/Akteur angeführten Standardargumente erfasst werden (siehe für Ausnahmen die nachfolgenden Hinweise).

Hinweis – mehrere Standardargumente gleichzeitig: Zu beachten ist, dass Aussagen von Sprechern/Akteuren prinzipiell *mehrere*, unterschiedliche *Standardargumente gleichzeitig* (z. B. in einem einzigen Satz) berühren können. In einem solchen Fall ist *jedes Standardargument gesondert und jeweils so präzise wie möglich* zu erfassen.

Hinweis – keine eindeutige Zuordnung von Standardargumenten: Es können Formulierungen vorkommen bei denen sich – auch aus dem Kontext des Artikels/Beitrags heraus – nicht eindeutig feststellen lässt, welches Standardargument durch eine Aussage angesprochen wird. Bei dieser Form von *Zweifelsfällen* sind *alle für den konkreten Fall möglichen* Standardargumente zu verschlüsseln. Beispiel: „Die FDP ist für eine weitere Aussetzung des Familiennachzugs bei der gleichzeitigen Möglichkeit von Härtefallregelungen. Die gänzliche Freigabe des Familiennachzuges ist angesichts der begrenzten Möglichkeiten bei Wohnen, Arbeit und Bildung aber aus Sicht der Partei nicht verantwortbar.“ Bei diesem Beispiel bleibt aufgrund der knappen Formulierung unklar, für wen die Konsequenzen nicht zu verantworten sind. Je nachdem, ob die Migranten selbst, die deutschen Behörden oder die Aufnahmegesellschaft gemeint ist, ergeben sich unterschiedliche Deutungen.

Hinweis – mehrmals Standardargument gleicher Ausprägung an verschiedenen Stellen eines Textes: Sofern ein Sprecher mehrmals ein Standardargument gleicher Ausprägung an verschiedenen Stellen eines Artikels oder Beitrags vorbringt bzw. Akteuren mehrmals das Standardargument gleicher Ausprägung zugewiesen wird, so ist das Standardargument diesbezüglich nur *einmal* zu verschlüsseln.

Hinweis – Gegenteilige bzw. gegensätzliche Deutungen: In der Übersicht von möglichen Deutungen finden sich einige Perspektiven, die Gegensätze darstellen (Beispiele: nationale Entscheidungshoheit in der Migrationspolitik vs. Entscheidungshoheit auf der supra- bzw. internationalen Ebene; Veränderung des politischen Diskurses in Richtung Autoritarismus vs. Libertarismus). Hier soll der Codierer darauf achten, ob die Befürwortung einer richtungspolitischen Deutung oder die Ablehnung der entgegengesetzten Deutung im Fokus steht (vgl. die Kategorie STA_VAL).

Liste möglicher Deutungsmuster und dazugehörige Codes

Kurzbezeichnung Deutungsmuster (Code)	Standardargumente	Zuordnung zu wertebezogenen Konfliktpolen	Quellen
**No Borders (01)**	Radikale, anarchistische Position der Grenzen- und in der Folge Staatenlosigkeit („no borders“).<br><br>Ausdrücklich allen Menschen, nicht etwa nur Arbeitsmigranten, sollte eine globale Bewegungs- und Niederlassungsfreiheit samt umfassenden Rechten im Aufenthaltsland unabhängig von ihrer Nationalität, Staatsbürgerschaft, ihrem Hintergrund et cetera zugestanden werden („Kein Mensch ist illegal.“).<br><br>Die Autorität von Staaten, hier insbesondere in migrationspolitischen Fragen, und Formen von Staatlichkeit werden allgemein in Frage gestellt. Bisweilen erfolgt eine Verknüpfung mit antikapitalistischen Haltungen.	Extrem integrativ im Sinne einer Ablehnung von Grenzen	Georgi (2016, 2015)<br><br>King (2016)
**Gesellschaftlicher Kosmopolitismus (02)**<br>Anmerkung: Aufgrund des seltenen Vorkommens im Untersuchungszeitraum wird diese Deutung im Rahmen der Auswertung dem Deutungsmuster „Multikulturalismus“ untergeordnet.	Das Denken in nationalistisch-ethnischen Grenzen wird als überholt angesehen.<br><br>Im Zentrum des Kosmopolitismus steht die Betonung der Gleichzeitigkeit und Vermischung verschiedener Zugehörigkeiten auf individueller	Integrativ im Sinne einer – über nationale Grenzen hinweg – kosmopolitischen Haltung	Eade und Ruspini (2014, 81f.) unter Verweis auf die Kosmopolitismus-Definition nach Beck und Grande (2007)<br><br>Eilders und Lüter (1998, 52f.)<br><br>Grande und Kriesi (2012, 12ff.)

	Ebene. Ausbildung einer (zusätzlichen) europäischen respektive globalen Identität.<br><br>Diesem Deutungstyp werden auch postmoderne Vorstellungen sowie die Konzeption postmigrantischer Gesellschaften zugeordnet. Darunter fällt etwa die Idee eines Konvivialismus: Demnach wird gesellschaftliches Zusammenleben als symmetrischer Lernprozess konzipiert, in dem sich alle Individuen und Teilgruppen einer (durch Migration geprägten) Gesellschaft permanent verändern und anpassen, um miteinander auszukommen.<br><br>Heutige durch Migrationsbewegungen geprägte Gesellschaften seien durch eine grundsätzliche „superdiversity“ (O’Brien 2016 mit Verweis auf Vertovec 2007) gekennzeichnet, die die Instabilität und Veränderbarkeit von Identitäten befördere.		Helbling (2014, 24ff.) – „multicultural identity frames“<br><br>Höglinger et al. (2012, 237ff.)<br><br>Münkler und Münkler (2016, 186f.)<br><br>O’Brien (2016, 301ff.)<br><br>Scheufele und Engelmann (2013, 539)<br><br>Voltmer (1999, 208f.; 1997, 192)

**Humanitarismus (03)**	Verständnis für und Bezüge auf die schwierige Situation von Migranten vor, während, nach der Migration: Thematisierung von Fluchtursachen, mangelnder Unterstützung, Formen der Diskriminierung, Verletzung von Menschenrechten, Ausbeutung, Verelendung von Migranten in Lagern; Ableitung von entsprechendem Handlungsbedarf.<br><br>Es soll eine menschenwürdige Behandlung und Unterbringung von Migranten in den Aufnahmestaaten gewährleistet sein.<br><br>Verweis auf Bewegungsfreiheit als Menschenrecht und die Befürwortung offener Grenzen: Bezüge auf die rechtliche Selbstbindung eines Landes sowie auf mögliche rechtliche Verstöße gegen Menschenrechtsabkommen (Europäische Menschenrechtskonvention, Artikel 13 der UN-Menschenrechtscharta) oder humanitäres Völkerrecht (Genfer Flüchtlingskonvention).<br><br>Die Pflicht der Hilfe und eines aktiven (ehrenamtlichen) Engagements seitens der Residenzgesellschaft erfolgt auf Basis des Gesellschaftsvertrages und zum Zwecke des Wohles aller Gesellschaftsmitglieder. Durch	Integrativ im Sinne eines (säkularen) Humanitarismus, grundsätzliche Ablehnung von (nationalistisch-völkischen) Tendenzen der Ausgrenzung	Arlt und Wolling (2016, Tab. 3)<br><br>Benson und Wood (2015, 807) – „Problems for immigrants"<br><br>Budde et al. (2018, 33ff.) –„Humanitätsframe"<br><br>Goedeke Tort et al. (2016, 509)<br><br>Münkler und Münkler (2016, 164ff.)<br><br>van Gorp (2005, 489ff.)

	die Aufnahme von Migranten werden humanitäre Haltungen in der Gesellschaft geweckt und gestärkt.		
**Barmherzigkeit (religiös) (04)**<br>Anmerkung: Aufgrund des seltenen Vorkommens im Untersuchungszeitraum wird diese Deutung im Rahmen der Auswertung der Deutung „Humanitarismus“ untergeordnet.	Die Pflicht der Hilfe und eines aktiven (ehrenamtlichen) Engagements seitens der Residenzgesellschaft oder spezifischer der Glaubensgemeinschaft erfolgt ausgehend von basalen, religiösen Geboten.	Integrativ im Sinne einer religiös motivierten Barmherzigkeit und Nächstenliebe, grundsätzliche Ablehnung von (nationalistisch-völkischen) Tendenzen der Ausgrenzung	Siehe Humanitarismus
**Multikulturalismus (05)**	Betonung kultureller Offenheit und des Mehrwerts kultureller Vielfalt („Melting Pot“-Metapher) für eine Gesellschaft – etwa im Sinne gesellschaftlichen Fortschritts. Das Zusammenleben ethnisch und kulturell unterschiedlicher Gruppen wird als Bereicherung eingeordnet.<br><br>Hervorgehobenes Ziel ist die friedvolle Koexistenz kulturell unterschiedlicher Gruppen in einer Gesellschaft.<br><br>Zusicherung von Eigenständigkeit und eigenen Rechten unterschiedlicher Kulturen und Bevölkerungsgruppen, die in einem Land zusammenleben.<br><br>Skeptische Haltung gegenüber restriktiven Bedingungen zum Erlangen umfassender staatsbürgerlicher Rechte.	Integrativ im Sinne von Toleranz, Akzeptanz bis hin zur Förderung von Multikulturalismus, Ablehnung von (nationalistisch-völkischen) Tendenzen der Ausgrenzung	Arlt und Wolling (2016, Tab. 3)<br><br>Benson und Wood (2015, 807) – „No problem“<br><br>Blätte (2017)<br><br>Eade und Ruspini (2014)<br><br>Eilders und Lüter (1998, 53)<br><br>Haller (2017a, 125)<br><br>Han (2010, 294ff.)<br><br>Helbling (2014, 24ff.) – „multicultural identity frames“, „moral-universal frames“<br><br>Höglinger et al. (2012, 237ff.)<br><br>O’Brien (2016, 295)

	Verweis auf moralische Prinzipien und universelle Rechte, die allgemein von jedem Menschen in gleicher Weise beansprucht werden können; Beispiele: Demokratie und Partizipation, Gleichheit, Gerechtigkeit, Rechtsstaatprinzip, Menschenrechte, bürgerliche Grundrechte, politische Rechte.		Scheufele und Engelmann (2013, 539)<br><br>Voltmer (1999, 209; 1997, 192)
	Akzeptanz von Mischformen alter und neuer Kultur (versus Illusion der vollständigen Assimilation).		
	Auffassung, dass ethnische und religiöse Gemeinschaften und Netzwerke „in der Fremde“ als „Sprungbrett“ oder „Übergangszonen“ in die Aufnahmegesellschaft fungieren können. Diese bieten Halt und Orientierung in noch unbekannten Ländern und Gesellschaften (Hoesch 2018, 179; Luft 2017, 108).		
	Multikulturalismus als Grundlage der Ausbildung gesellschaftlicher Identität: „Kulturelle Identität bedeutet, dass unterschiedliche Menschen zwar örtlich gemeinsam leben, aber ihre jeweils eigenen Charakteristika bewahren. Dies kann seinen Ausdruck in eigenen Institutionen und		

	Rechtsansprüchen (Schulen, explizite Schutzrechte) finden.“ (Eilders und Lüter 1998, 53)		
**Toleranz (religiös) (06)**<br>Anmerkung: Aufgrund des seltenen Vorkommens im Untersuchungszeitraum wird diese Deutung im Rahmen der Auswertung dem Deutungsmuster „Multikulturalismus“ untergeordnet.	Betonung von Offenheit gegenüber anderen Religionen und Forderung eines friedlichen Miteinanders sowie interreligiösen Dialogs zum Zwecke der Integration.<br><br>Skeptische Haltung gegenüber pauschalen Fehldarstellungen anderer Religionen („Muslime sind alle gewalttätig.“), deren Ausgrenzung und Diskriminierung.<br><br>Die Pflicht zur Toleranz folgt in dieser Deutung explizit basalen, religiösen (nicht zwingend nur christlichen) Geboten.	Integrativ im Sinne einer religiös motivierten Nächstenliebe beziehungsweise einer Toleranz gegenüber anderen Religionsgemeinschaften, grundsätzliche Ablehnung von Tendenzen der Ausgrenzung von anderen Religionsgemeinschaften	Siehe Multikulturalismus
**Republikanismus (liberal assimilationism) (07)**	Republikanische Konzeption eines „liberal assimilationism“ (O’Brien 2016; Joppke und Morawska 2003) als Integrationsmechanismus und als Grundlage gesellschaftlicher Identität. Eingeforderte Verpflichtung der Migranten, die universellen Menschenrechte und demokratischen Grundwerte zu achten und sich diese anzueignen. Im Zuge dieser Deutung wird bisweilen gefordert, dass die Sprache der Mehrheitsgesellschaft von Migranten als Grundlage der Integration erlernt wird.	Moderat liberal/integrativ im Sinne des vorrangigen Schutzes individueller Freiheitsrechte und demokratischer Grundwerte	Han (2010, 337)<br><br>Hoesch (2018, 152f.)<br><br>Joppke und Morawska (2003, 5ff.)<br><br>Koopmans (2017, 194ff.)<br><br>Luft (2017, 106ff.)<br><br>Münkler und Münkler (2016, 288f.)<br><br>O’Brien (2016, 292ff.)

Dem „liberal assimilationism“ liegt das Ziel zugrunde, dass die individuelle Autonomie aller Gesellschaftsmitglieder nicht gefährdet werden darf.

Republikanische Formen staatsbürgerlicher Identität im Sinne eines Verfassungspatriotismus.

Religionskritik im Sinne einer Herausforderung für demokratische Werte der Aufklärung (zum Beispiel Verhältnis Kirche-Staat, Rechtsstaatlichkeit, Meinungs- und Religionsfreiheit, Geschlechtergerechtigkeit); Häufiges Beispiel ist das Frauenbild im Islam:

- Frauen als Opfer einer frauenfeindlichen Religion
- Kopftücher als Symbol der Unterdrückung
- „kulturell bedingte Gewalt“ gegen Frauen (häusliche Gewalt, Ehrenmorde, Genitalverstümmelung)
- Interpretation des Ehrbegriffs und Patriarchalismus
- Praxis einer „Paralleljustiz“

Ein weiteres Beispiel sind feindliche Haltungen auf Seiten der Migranten gegenüber anderen Religionen (etwa Antisemitismus im Islam).

**Assimilation (08)**	Die Integration von Migranten in die Gesellschaft funktioniere nach dieser Perspektive nur unter der Bedingung, dass sie sich an die im Aufnahmeland geltenden Gesetze, sozialen Normen, Umgangsformen und Traditionen halten.<br><br>Assimilation meint hier, dass kulturelle Gruppen sich der Leitkultur der Aufnahmegesellschaft anpassen. Sie sollen diese übernehmen, das heißt sie sollen sich kulturell und damit über geteilte demokratische Grundprinzipien hinaus assimilieren.<br><br>Die „Leitkultur" des Aufnahmelandes – etwa in Anlehnung an christliche und aufklärerische Werte, Religionen und Kultur des „Abendlandes" – fungiert als Grundlage der Ausbildung einer nationalen Identität. Die in multikulturalistischen Überlegungen betonte Eigenständigkeit der anderen Kulturen (eigene Sprache, Schulen) wird abgelehnt. Einforderung des Austausches mit der Mehrheitskultur auch in interpersonalen Netzwerken, Freundeskreisen et cetera.	Abgrenzend-autoritär im Sinne der Forderung nach Anpassung an Gesetze, Regeln und insbesondere an kulturelle Traditionen der Aufnahmegesellschaft (strenge Interpretation des Gedankens der Assimilation)	Arlt und Wolling (2016, Tab. 3)<br><br>Benson und Wood (2015, 807) – „Problems for society"<br><br>Eade und Ruspini (2014)<br><br>Eilders und Lüter (1998, 53)<br><br>Grande und Kriesi (2012, 12ff.)<br><br>Hainmueller und Hopkins (2014, 230ff.) – „Sociotropic Threats"<br><br>Han (2010, 294ff.)<br><br>Helbling (2014, 24ff.) – „nationalistic frames"<br><br>Hoesch (2018, 82ff.)<br><br>Höglinger et al. (2012, 237ff.)<br><br>Koopmans (2017, Kap. 1)<br><br>Roggeband und Vliegenthart (2007, 531) – „Islam-as-Threat Frame"<br><br>Scheufele und Engelmann (2013, 539)

	Problematisierung: Ohne eine „Leitkultur“ bestehen womöglich nur schwer oder nicht zu überbrückende, kulturelle und religiöse Differenzen im Alltag zwischen Migranten sowie der Aufnahmegesellschaft.		
**Utilitarismus Aufnahmegesellschaft (09)**	Man geht im Rahmen dieser Deutung davon aus, „dass der soziale Zusammenhalt durch die heftige Abwehr eines Teils der Gesellschaft gefährdet und so auch das gelebte Grundrecht bedroht sei“ (Haller 2017a, 125). Man begründet „diese Einschätzung mit dem aufbrechenden Fremdenhass, dem Erfolg rechtsnationaler Populisten und der zunehmenden Straßengewalt“ (ebd.). Diese Tendenzen werden als Kennzeichen dafür interpretiert, „dass die Zahl der Flüchtlinge an die Grenzen der Sozialverträglichkeit gelangt sei“ (ebd.) und damit auch die Chancen auf eine Integration der Migranten in die Gesellschaft verringert seien.<br><br>Bei diesem Deutungsmuster finden sich Argumentationen im Sinne einer Güterabwägung, „dass die Sicherung des sozialen Zusammenhalts ein höheres Gut sei als die Aufnahme immer neuer Flüchtlinge“ (ebd.).	Implizit abgrenzend beziehungsweise nationalistisch im Sinne des Vorrangs des sozialen Zusammenhalts in der Aufnahmegesellschaft	Haller (2017a, 125)<br><br>Helbling (2014, 24ff.) – „pragmatic frames“<br><br>Höglinger et al. (2012, 237ff.)

**Anti-Egalitarismus und Ethnopluralismus (10)**	Grundannahme: Pauschale Differenzierung der Weltbevölkerung nach kulturellen Räumen, wobei jedes Individuum einer (homogenen) Kultur zugeordnet werden kann. Kultur wird hierbei nicht als soziale Praxis, als Habitus oder Konvention betrachtet, sondern als „natürliches“ Merkmal eines Individuums qua zugeschriebener Zugehörigkeit zu einem statischen Kollektiv. Dieses Kollektiv kann ein durch eine gemeinsame Kultur definiertes Volk oder eine Ethnie, Nation, Religion (Islam, Judentum) sowie größere geographische Räume (Maghreb, naher Osten, Arabien, Afrika) meinen.<br><br>Zentrale Vorstellung der Unvereinbarkeit: Prognose von Konkurrenzverhältnissen und Konflikten zwischen nicht zu vereinbarenden „eigenen“ und „fremden“ Kulturkreisen („clash of cultures“; „clash of civilizations“); Darstellung von anderen Kulturen und Ethnien als Bedrohung für die einheimische Bevölkerung; vorrangiges Ziel der Bewahrung einer kulturellen Homogenität beziehungsweise von kulturell homogenen Gesellschaften.	Extrem abgrenzend beziehungsweise nationalistisch im Sinne einer klar segregierenden und völkisch-ethnozentrischen Haltung	Carter (2017)<br><br>Kitschelt (1995)<br><br>Koopmans et al. (2005, 11f.)<br><br>Künzel (2007, 157ff.)<br><br>Küpper et al. (2016)<br><br>Mudde (2017)<br><br>Winkler (2001)<br><br>Zick et al. (2016)

	Übertragung der in der Grundannahme angelegten Differenzsemantik „eigen versus fremd“ (Udris 2011, 57, 60ff.) als Maßstab legitimer Politik: Differenzierung einer ursprünglichen „Volksgemeinschaft“ mit vollen und einer Minderheit von Fremden mit deutlich eingeschränkten politischen, sozialen und kulturellen Rechten („Vorrechte von ethnisch Deutschen“, „Muslime haben nicht das Recht im christlich geprägten Deutschland zu leben.“).<br><br>Präferenz eines segregierenden Ethnopluralismus, um Verluste von Identität und sozialer Kohäsion („Gefährdung des friedlichen Miteinanders“) durch ethnisch-kulturelle Vermischung zu vermeiden (Argumentation der Neuen Rechten): Befürwortung eines Programms der ethnokulturellen Revision, Aufhebung der seit den 1970er Jahren einsetzenden, gesellschaftlichen Vielfalt und Durchmischung im Sinne ethnisch-kultureller Grenzziehungen zur Definition einer Nation; Befürwortung von politischen Maßnahmen, um die Vermischung von homogenen Gruppen zu unterbinden oder rückgängig zu machen und die „Reinheit“ von Völkern/Ethnien/Nationen zu gewährleisten.	

**Innere Sicherheit: präventiv/integrativ (11)**	Bei dieser Deutung wird die Verknüpfung von Migrationsbewegungen und steigender Kriminalität bestritten. Stattdessen wird auf andere Zusammenhänge aufmerksam gemacht, die kriminologisch ermittelt werden können. Dazu gehören etwa problematische Aspekte wie die soziale und ökonomische Perspektivlosigkeit von Migranten, Lagerkoller sowie das Aufeinandertreffen von Menschen mit religiös-kulturellen Differenzen in Aufnahmezentren und/oder Gewalt gegen beziehungsweise zwischen Migranten. Häufig verbunden mit der Forderung nach einer Verbesserung der Situation von Migranten sowie nach eher präventiven Maßnahmen zur Verbesserung der inneren Sicherheit.	Integrativ im Sinne einer Befürwortung präventiver Maßnahmen zur Gewährleistung innerer und persönlicher Sicherheit	Koopmans (2017, 142ff.)<br><br>Mediendienst Integration (2018)<br><br>Pfeiffer et al. (2018)
**Innere Sicherheit: Law-and-Order (12)**	Im Rahmen dieser Deutung wird die Zuwanderung von Migranten mit dem Verlust von Ordnung, innerer Sicherheit, zunehmender Kriminalität bis hin zur Angst vor terroristischen Akten verknüpft.<br><br>Häufig verbunden mit der Forderung nach einer strengen Law-and-Order-Politik und einer rigiden Abschiebepraxis.	Abgrenzend-autoritär im Sinne einer Law-and-Order-Haltung zur Gewährleistung innerer/gesellschaftlicher und persönlicher Sicherheit	Benson und Wood (2015, 807) – „Problems for society“<br><br>Budde et al. (2018, 33ff.) – „Law-and-Order-Frame“<br><br>Goedeke Tort et al. (2016, 508)<br><br>Hainmueller und Hopkins (2014, 230ff.) – „Sociotropic Threats“<br><br>Helbling (2014, 24ff.) – „security frames“

**Sozialstaat als Integrationsinstrument (13)**	Für das Gelingen einer Integration von Migranten gilt es, über sozialstaatliche Maßnahmen deren Teilhabe an der Gesellschaft sicherzustellen.<br><br>Gewährleistung von Integration und Identitätsbildung von Migranten gerade durch Teilhabe am Sozialstaat.	Gemeinwohlorientiert im Sinne einer Vorstellung von (sozial-)staatlichen Leistungen als Integrationsinstrument	Hoesch (2018, 155ff.)<br><br>Münkler und Münkler (2016, 112f.)
**Ökonomischer Nutzen (14)**	Wertebezogene Aussagen, die in der Migration einen ökonomischen Nutzen (potenzielle, kostengünstige oder hochqualifizierte Arbeitskräfte, Fachkräfte, Steigerung der Produktivität und Wettbewerbsfähigkeit) sehen.<br><br>Befürwortung offener Grenzen als Verlängerung eines freien, gemeinsamen Wirtschaftsraums zum Zwecke eines langfristigen Wirtschaftswachstums und der Vermehrung von Wohlstand (Beispiel: Freizügigkeit innerhalb des Schengen-Raums der Europäischen Union).<br><br>Einforderung einer „neoliberalen Wende“ der Migrationspolitik.<br><br>Kritik unter anderem an der unzureichenden Flexibilität des Arbeitsmarktes und des Sozialstaates beziehungsweise an staatlichen Regulierungsinstrumenten; Verknüpfung mit	Marktliberal im Sinne einer Gewinn-, Wachstums- und Effizienzorientierung	Arlt und Wolling (2016, Tab. 3)<br><br>Blätte (2017, 168)<br><br>Benson und Wood (2015, 807) – „No problem“<br><br>Goedeke Tort et al. (2016, 508f.)<br><br>Haller (2017a, 125)<br><br>Helbling (2014, 24ff.) – „economic frames“<br><br>Hoesch (2018, 158) unter Verweis auf Ruhs und Martin (2008)<br><br>Höglinger et al. (2012, 237ff.)<br><br>Koopmans (2017, 20ff.)

	der Forderung nach Deregulierung und Flexibilisierung; auch: schlanker Sozialstaat als besonderer Anreiz für Hochqualifizierte und Gefahr ungewollter Effekte des Sozialstaates für Niedrigqualifizierte (so genannte „Wohlfahrtsfalle“).<br><br>Erörterung von Trade-Off-Beziehungen zwischen den Migranten zugestandenen Rechten und Leistungen sowie der erlaubten Zahl an Zuwandernden (ökonomische Kosten-Nutzen-Erwägungen).<br><br>Ökonomisch-effizienzorientierte Perspektive des Diversity Management in Unternehmen und Behörden (kulturelle Vielfalt als ökonomisch gewinnbringende Ressource).		
**Ökonomisch nützliche Migrationstypen (15)**	In dieser Deutung wird argumentiert, dass ausschließlich diejenigen Migranten aufgenommen und integriert werden sollten, die einen Nutzen für die wirtschaftliche Entwicklung des Landes darstellen.<br><br>Darunter auch Kritik: Die momentanen Migranten entsprechen nicht denjenigen, die der Arbeitsmarkt des Aufnahmelandes dringend benötigt.<br><br>In weniger radikaler Form als bei populistischen Akteuren entspricht	Synthese einer marktliberal-gewinnorientierten und abgrenzenden Perspektive im Sinne einer Differenzierung von „marktrelevanten“ Migrationstypen	Münkler und Münkler (2016, 94ff.)<br><br>Schammann (2017, 150f.)

	diese Deutung der am ökonomischen Nutzen einzelner Personen orientierten und stark gesteuerten Migrationspolitik von Staaten wie etwa den USA, Kanada, Neuseeland oder Australien (Hoesch 2018, 165f.; Han 2010, 170ff.).		
**Kosten und Belastung des Sozialstaats (16)**	Deutungen, die dieser Kategorie zugeordnet werden, sind dadurch gekennzeichnet, dass sie aus einer Kostenperspektive insbesondere die Belastung des Sozialstaats durch Zuwanderung fokussieren:<br><br>Betonung einer zunehmenden Überlastung des – ohnehin schon tiefgreifenden – Wohlfahrtsstaats aufgrund von Migration.<br><br>Migranten als besondere Herausforderung aufgrund ihrer geringen finanziellen Unabhängigkeit.<br><br>Verweise auf Kosten für die Allgemeinheit, die die positive ökonomische Entwicklung des Landes gefährden könnten, sowie auf die mögliche Verschwendung staatlicher, finanzieller Mittel für Menschen mit einer unsicheren Bleibeperspektive.	Marktliberal im Sinne einer Orientierung an ökonomischer Stabilität und an der Effizienz staatlichen Handelns, Ziel einer möglichst geringen Belastung des Sozialstaats	Arlt und Wolling (2016, Tab. 3)<br><br>Benson und Wood (2015, 807) – „Problems for society“, „Cause pull factors“<br><br>Goedeke Tort et al. (2016, 509)<br><br>Hainmueller und Hopkins (2014, 227ff.) – „Fiscal burden“, „Labor market competition“<br><br>Helbling (2014, 24ff.) – „labour and social security frames“<br><br>Höglinger et al. (2012, 237ff.)

**Wohlfahrtschauvinismus (17)**	Diese Kategorie bezieht sich auf sozialautoritäre Deutungen, bei denen die Belastung des Sozialstaats entlang ethnischer Grenzziehungen verhandelt wird. Solche Grenzziehungen fungieren als Maßstab legitimer Sozialpolitik.<br><br>Aussagen beziehen sich hier auf die Vorstellung einer ausgrenzenden Konkurrenz um Anspruchsberechtigung nach dem Muster „Sozialleistungen für Migranten bedeuten den Abzug von Sozialleistungen für uns Deutsche.“. Die sozialstaatliche Belastung wird gedeutet als problematische Einschränkung von Rechten, die man als (deutscher) Volksgenosse erworben hat (Ethnisierung des Problems). Damit verbunden sind in der Konsequenz auch Forderungen nach einer geringeren wohlfahrtsstaatlichen Unterstützung für Migranten im Vergleich zur Unterstützung der einheimischen Bevölkerung.<br><br>Neben einer Konkurrenz um sozialstaatliche Leistungen können im Rahmen dieser Deutung auch andere Bezugspunkte beziehungsweise soziale Felder der Konkurrenz herangezogen werden. Beispiel des Arbeitsmarktes: Hier wird die zusätzliche	(Sozialstaatlich-)Abgrenzend im Sinne der Vorenthaltung von Rechten für Minoritäten	Benson und Wood (2015, 807) – „Problems for society“<br><br>Hainmueller und Hopkins (2014, 227ff.) – „Fiscal burden“, „Labor market competition“<br><br>Helbling (2014, 24ff.) – „labour and social security frames“<br><br>Höglinger et al. (2012, 237ff.)<br><br>Kitschelt (1995)<br><br>Münkler und Münkler (2016, 114ff.)

	Konkurrenz auf dem Arbeitsmarkt durch Migranten thematisiert und mit Ängsten vor einem gedrückten Lohnniveau, vor Arbeitslosigkeit und Armut der Mitglieder der Aufnahmegesellschaft verknüpft. Eingefordert wird in diesem Zusammenhang zumeist eine starke Einschränkung bis hin zu einer Verwehrung des Zugangs von Migranten zum einheimischen Arbeitsmarkt.<br><br>Im Rahmen dieser Deutung wird bisweilen die Differenzierung von „Wirtschaftsflüchtlingen“, „Armutsmigranten“, „Sozialtouristen“ einerseits und „echten“ politischen Flüchtlingen andererseits zur Begründung herangezogen.		
**Internationale Kooperation (18)**	Die grenzüberschreitende Kooperation von Nationalstaaten und Regionen (und beispielsweise auch Nichtregierungsorganisationen) sei zur Bearbeitung globaler Herausforderungen wie umfassenden Migrationsströmen die effizientere und damit überlegene Form.<br><br>Ziel sind friedliche Kompromisslösungen und die Vertiefung grenzüberschreitender Zusammenarbeit. Voraussetzung der Zusammenarbeit ist internationale Solidarität zwi-	Integrativ im Sinne der Entscheidungsfindung, gemeinsamen Problemlösung und Lastenteilung auf supranationaler Ebene	Grande und Kriesi (2012, 12ff.)<br><br>Luft (2017, 117ff.)<br><br>Münkler und Münkler (2016, 213ff.)

	schen den verschiedenen Nationalstaaten. Zudem sollten supra- beziehungsweise internationale Organisationen im Sinne einer Ausweitung von Aufgaben und Verantwortlichkeiten gestärkt werden.<br><br>Kritik an einer möglichen Rückkehr zu einzelstaatlichen Lösungen (Grenzschließung, Widerstand gegenüber Verteilungsschlüsseln et cetera).		
**Nationalstaatliche Souveränität (19)**	Bewahrung nationaler Souveränität und die Vertretung nationaler Interessen und Rechte im Rahmen der Lösung von Problemen. Auf den Nationalstaat ausgerichtete Orientierung in der Entscheidungsfindung aus nutzenbezogenen („höhere Effektivität nationaler Lösungen“) und symbolischen („Deutschland als mächtiger Staat und starke Nation in der Weltpolitik“) Gründen.<br><br>Verweise auf einen möglichen Steuerungsverlust von Nationalstaaten („Entgrenzung“) mit dem Ziel der Rückkehr zu einzelstaatlichen Problemlösungen.	Nationalistisch-abgrenzend im Sinne der Bewahrung nationaler Souveränität in der Entscheidungsfindung	Grande und Kriesi (2012, 12ff.)<br><br>Hoesch (2018, Kap. 4.3.5, Kap. 4.5, Kap. 4.6)<br><br>Münkler und Münkler (2016, 213ff.)

**Effektivität des Regierens (20)**	Hierunter fallen relativ ähnliche Perspektiven, die in den konkreten Fällen mehr oder wenig stark autoritär geprägt sein können. Zum Teil, aber nicht immer, wird diese Deutung als pragmatisches Argument zur Rechtfertigung einer ausgrenzenden Haltung genutzt. Fokussiert wird die Kapazität staatlicher sowie supranationaler Institutionen effektiv zu handeln.<br><br>Hervorhebung von Fragen der rechtlichen Umsetzung und der hohen Bedeutung von Rechtssicherheit (wie bei Verweisen auf rechtliche Grauzonen, den unklaren Status von Asylbewerbern oder die Zahl illegaler Migranten).<br><br>Befürchtungen, dass die staatlichen Institutionen beziehungsweise das juristische Regulationssystem im Aufnahmeland – sowie auf internationaler Ebene –nicht in der Lage seien, die hohen und aufgrund von „Kettenwanderungen“ vermutlich auch kontinuierlichen Flüchtlingszahlen zu bewältigen. Erwartung eines „Systeminfarkts“ (Luft 2017, 88 unter Verweis auf Klos 2013) als mittelfristige Konsequenz. Ziel ist die „realistische“ Orientierung am Prinzip der Machbarkeit.	Moderat autoritär-abgrenzend im Sinne von Effektivität des Regierens als Eigenwert	Benson und Wood (2015, 807) – „Problems for authorities“<br><br>Budde et al. (2018, 33ff.) – „Maßnahmenframe“<br><br>Haller (2017a, 125)<br><br>Helbling (2014, 24ff.) – „pragmatic frames“<br><br>Höglinger et al. (2012, 237ff.)<br><br>Luft (2017, 97ff.)

**Kosteneffiziente Verwaltung (21)**	Spezifische Kosten-Perspektive: Kritik an zeitraubenden, bürokratischen Strukturen, die einer kosteneffizienten Lösung migrationspolitischer Herausforderungen im Wege stehen.	Übertragung einer marktliberalen Haltung auf administrative Fragen einer kosteneffizienten Verwaltung	Benson und Wood (2015, 807) – „Problems for authorities“<br><br>Helbling (2014, 24ff.) – „pragmatic frames“
**Linke Elitenkritik (22)**	Der politischen Elite wird vorgeworfen, dass zu wenige oder kontraproduktive Maßnahmen für die umfassende Unterstützung und Integration von Migranten getroffen werden.<br>Das System zur Regulierung von Migration wird als zu restriktiv oder inhuman bewertet. Das Handeln der politischen Elite sorge für eine Verschlechterung der Situation von Migranten.<br><br>Sonderform der Elitenkritik gegenüber der Europäischen Union: Vorwurf einer „bigotten“ Politik, in deren Rahmen innerhalb der EU Bewegungsfreiheit möglich ist, während sie sich nach außen durch ein rigides Grenzregime abschottet (Metapher der „Festung Europa“).<br><br>Kritik an der Ungerechtigkeit des EU-Asylregimes, das den Tod vieler Menschen während der Flucht in Kauf nehme und die Angewiesenheit der Flüchtenden auf „Schlepperbanden“ verstärke; damit werde als Nebenprodukt ungerechterweise auch	Integrativ(-solidarisch)	Benson und Wood (2015, 807) – „Cause system (pro-immigration)“<br><br>Han (2010, 196f.)<br><br>Hoesch (2018, Kap. 7)<br><br>Koopmans (2017, Kap. 3)<br><br>Münkler und Münkler (2016, 209, 216f.)<br><br>Philo et al. (2013, Kap. 2)<br><br>Udris (2011, 62f.)

	eine spezifische Gruppe von Migranten, das heißt junge, gesunde Männer, die eine Flucht finanzieren können, bevorzugt (Koopmans 2017, Kap. 3).<br><br>Erklärung der „Migrationskrise“ als Folge oder Ausdruck anderer Konflikte und Herausforderungen:<br>Kritik an autoritären Regimen und Unterstützung solcher Regime etwa durch Waffenlieferungen seitens westlicher Demokratien; Folgen des Neoliberalismus und soziale Ungleichheit als Kernprobleme; rechter Populismus/Extremismus; Entstehung von „Parallelgesellschaften“ nicht primär religionsbedingt, sondern als Folge von staatlicher Wohnungspolitik und sozioökonomischer Benachteiligung; Radikalisierung von Migranten als Konsequenz von Diskriminierung.	

**Moderat autoritäre Elitenkritik (23)**	Der politischen Elite wird beim moderat autoritären Deutungstyp vorgeworfen, dass a) die Herausforderungen und Probleme, die mit der Zuwanderung von Fremden verknüpft sind, von ihr nicht verhindert oder zu wenig entschieden behandelt werden (Vorwurf der Untätigkeit). Die aktuellen Maßnahmen zur Regulierung der Migration auf nationaler, europäischer und internationaler Ebene werden als zu lasch und/oder als inadäquat bewertet.<br>Teil dieses moderat autoritären Deutungstyp kann zudem b) die Thematisierung von Überforderung, Unsicherheiten bis hin zu Ahnungslosigkeit und anschließenden Fehlentscheidungen auf der behördlichen Vollzugsebene sein.	Moderat abgrenzend-autoritär	Benson und Wood (2015, 807) – „Cause system (anti-immigration)"<br><br>Münkler und Münkler (2016, 223ff.)
**Extrem autoritäre Elitenkritik (24)**	Ausgehend von im Anti-Egalitarismus prominenten Feindbildern wendet sich die Feindschaftserklärung im Rahmen dieser Deutung gegen diejenigen, die die Anwesenheit der Feinde im Innern zulassen, das heißt gegen die politische Elite.<br><br>Die „bedrohte Volksgemeinschaft" werde in zentralen Entscheidungen nicht berücksichtigt und ihre Situation verschlechtere sich durch das Handeln der politischen Elite. Das Volk könne nur durch eine bessere	Extrem abgrenzend-autoritär	Benson und Wood (2015, 807) – „Cause system (anti-immigration)"<br><br>Carter (2017)<br><br>Künzel (2007)<br><br>Küpper et al. (2016)<br><br>Mudde (2017)<br><br>Münkler und Münkler (2016, 201ff.)<br><br>Stöss (2010)

	Führung gerettet werden, weshalb ein Austausch der Elite eingefordert wird.		Udris (2011, 63, Fn. 46)<br><br>Zick et al. (2016)
	Bisweilen werden im Rahmen dieser Deutung verschwörungstheoretische Auffassungen vorgebracht. Es handelt sich dabei um Aussagen, in denen negative politisch-gesellschaftliche Entwicklungen unter Verweis auf (empirisch nicht prüfbare) Verschwörungen erklärt werden. Der politischen Elite wird vorgeworfen, dass die Probleme, die mit der Zuwanderung von Fremden verknüpft sind, von ihr bewusst verschleiert werden oder sogar gewollt sind. Der Elite wird hinsichtlich der Migrationspolitik ein konspiratives Handeln unterstellt, Beispiel: „Hinter dem Handeln der deutschen Regierung steckt der langfristige Plan, in Deutschland einen muslimischen Staat zu errichten.“.		
	Extremer Aktivismus als mögliche praktische Fortsetzung der Elitenfeindschaft: Physische Gewalt und terroristische Akte (gegen Migranten und/oder gegen die politische Elite) werden als legitime Mittel zur Durchsetzung der eigenen politischen Position angesehen. Unterstüt-		

	zung oder sogar Substitution der Polizei durch „Bürgerwehren“ (Vigilantismus).		
**Polarisierung der Gesellschaft (25)**	Die „Flüchtlingskrise“ und der Migrationsdiskurs werden als Auslöser für eine allgemeine Polarisierung der Gesellschaft angesehen.	Kein Bezug zu Konfliktpolen	Benson und Wood (2015, 807) – „Problems for society“<br><br>Budde et al. (2018, 33ff.) –„Konfliktframe“
**Politische Kultur nach links (26)**	Der Migrationsdiskurs verdeutlicht die Dominanz von Akteuren und Meinungen aus dem linken politischen Lager.<br><br>Vorwürfe eines „Meinungsdiktats“ des „links-grünen Mainstreams“ (linke kulturelle Hegemonie).<br><br>Kritik an überzogener „political correctness“ im politischen Diskurs.<br><br>Der Migrationsdiskurs lässt erkennen, dass in der Asylpolitik zunehmend liberalisierende Policy-Vorschläge seitens etablierter Parteien vorgebracht und beschlossen werden.	Abgrenzend	Benson und Wood (2015, 807) – „Problems for society“<br><br>Budde et al. (2018, 33ff.) –„Konfliktframe“<br><br>Küpper et al. (2016)
**Politische Kultur nach rechts (27)**	Der Migrationsdiskurs verdeutlicht die Verrohung der gesellschaftlichen Debatte durch Akteure und Meinungen aus dem rechten Lager.<br><br>Hinweise auf die Gefahr eines erstarkenden Rechtspopulismus und Rechtsradikalismus.	Integrativ	Benson und Wood (2015, 807) – „Problems for society“<br><br>Budde et al. (2018, 33ff.) –„Konfliktframe“

	Der Migrationsdiskurs lässt erkennen, dass in der Asylpolitik zunehmend restriktive Policy-Vorschläge – womöglich als Reaktion auf die steigende Popularität rechter Bewegungen und Parteien – seitens etablierter Parteien vorgebracht und beschlossen werden.<br><br>Zudem wird das Ausmaß (latenter) diskriminierender bis fremdenfeindlicher Haltungen in großen Teilen der Gesellschaft inklusive der Eliten deutlich.	

Deutungsmuster: Valenz (STA_VAL)

Hier wird zusätzlich zur Identifikation von Deutungsmustern pro wertebezogener Aussage festgehalten wie Sprecher sich gegenüber der jeweils identifizierten, wertebezogenen Aussage positionieren bzw. welche Positionierung anderen Akteuren zugewiesen wird. Es geht um die Prüfung, ob Sprecher/Akteure als *Befürworter bzw. Garant* oder als *Gegner* der mit Standardargumenten ausgedrückten Perspektive fungieren.

Grundsätzlich ist eine Wertung definiert als die Abgabe eines subjektiven Urteils bezüglich des Wertes eines Objektes oder Subjektes durch einen Urteilenden. Ein verbalisiertes Urteil ist ein Satz, der ausdrücklich eine Wertung beinhaltet oder eine solche impliziert. Explizite Wertungen können sprachlich durch Wertbegriffe wie „gut“ – „schlecht“, „gerecht“ – „ungerecht“, „sinnvoll“ – „sinnlos“ etc. signalisiert werden (z. B. „Aus Sicht der SPD ist die Rückbesinnung auf gesellschaftliche Solidarität in der jetzigen Krisensituation besonders sinnvoll.“, explizite Befürwortung; „Zwischen Migranten erster und zweiter Klasse zu unterscheiden ist aus Sicht der Grünen vollkommen unangebracht.“, explizite Ablehnung).

Prominent ist die Form einer impliziten Wertung bzw. einer impliziten Positionierung als Garant für die Durchsetzung bestimmter wertebezogener Grundhaltungen. Diese Form kann insbesondere dann auftreten, wenn wertebezogene Aussagen als Rahmen von Policy-Vorschlägen genutzt werden. Beispiel: „Die SPD plädiert für die Möglichkeit des Familiennachzugs, weil der Nachzug ein wichtiges Element zur Integration der Migranten ist“. Bei dieser Form findet sich zwar keine explizite Befürwortung und dennoch wird durch die Art der Formulierung deutlich, dass die SPD sich als Garant von (sozial)staatlichen Maßnahmen zur Integration positioniert. Das folgende Beispiel soll demgegenüber die implizite Positionierung eines Sprechers/Akteurs als Gegner einer angesprochenen Grundhaltung illustrieren: „Mit der jahrelangen Politik der Bemutterung ethnischer Minderheiten muss gebrochen werden, so der Experte. Der Ansporn zur Eigeninitiative werde dadurch unterdrückt.“

Für die Codierung als ambivalente Bewertung ist eine Abwägung verschiedener Positionierungen notwendig. Beispiel: „Die Ausrichtung der CDU bleibt unklar. Zum einen will man seinem christlich-solidarischen Profil treu bleiben. Zum anderen werden die parteiinternen Forderungen nach einer deutlich restriktiveren Positionierung in der Flüchtlingspolitik immer lauter.“

Neben verbalisierten Wertungen können in Videosequenzen auch indirekte Wertungen angewendet werden. Diese zeigen sich durch Mimik, Gestik, oder Tonfall (z. B. im Falle von Ironie), nicht jedoch durch eine explizite Aussage. In europäischen Kulturen bedeutet bezüglich der

Mimik etwa die Stirn zu runzeln Tadel, die Unterlippe vor zu schieben und die Augen zu verdrehen Ungläubigkeit bzw. Skepsis, die Nase zu rümpfen und die Nasenlöcher zu blähen Abscheu bzw. Ekel und die Züge „versteinern“ zu lassen Ablehnung.

1 = Zustimmung: Standardargument wird befürwortet bzw. Akteuren wird eine Befürwortung der dazugehörigen Deutung zugeschrieben. Sprecher/Akteure positionieren sich als Garant für die Durchsetzung bestimmter Grundhaltungen.

2 = Ambivalenz: Standardargument wird vom Sprecher ambivalent bewertet bzw. Akteuren wird eine ambivalente Bewertung der dazugehörigen Deutung zugeschrieben. Sprecher/Akteure haben keine feste Position gegenüber den angesprochenen Grundhaltungen.

3 = Zurückweisung: Standardargument wird vom Sprecher abgelehnt bzw. Akteuren wird eine Ablehnung der dazugehörigen Deutung zugeschrieben. Sprecher/Akteure positionieren sich als Gegner für die Durchsetzung bestimmter Grundhaltungen.

Sprecher und Akteure – Vorbemerkung und übergeordnete Hinweise

Problembezogene Aussagen werden von Sprechern geäußert bzw. Akteuren zugeordnet. Sprecher und Akteure werden mit einer *gemeinsamen* Kategorie erfasst. Als *Sprecher* gelten in Anlehnung an Haller (2017b) identifizierbare Aussageträger im Text, die als Quellen von problembezogenen Aussagen *im Artikel/Beitrag explizit* angegeben sind. Sprecher können vor allem dadurch identifiziert werden, dass sie direkt oder indirekt zitiert werden. *Akteure* sind im Text identifizierbare Handlungsträger. Sie sind dementsprechend durch Verben des Handelns zu erkennen. Auch anhand der Beschreibung eines bestimmten Handelns von Akteuren lassen sich deren problembezogene Haltungen ablesen und codieren. Beispiele: „Die Grünen verlangen eine angemessene Unterbringung der Asylsuchenden, um die humanitäre Verpflichtung Deutschlands zu erfüllen.“, „Die Kanzlerin appellierte an die EU-Mitgliedsstaaten, sich am Grundsatz der Solidarität zu orientieren.“. Grundsätzlich sind Sprecher und Akteure so präzise wie möglich zu codieren, notfalls sind die aufgeführten Oberkategorien zu verwenden.

Hinweise zur Verknüpfung von Sprechern/Akteuren und problembezogenen Aussagen innerhalb eines Textes:

Verschlüsselung von Beitragsautoren als Sprecher/Akteur: Finden sich in einem Text problembezogene Aussagen, die allerdings keinem Sprecher/Akteur im definierten Sinne zugeordnet werden können (z. B. insbesondere bei der Stilform ‚Kommentar‘), so wird der *Beitragsautor* bzw. die *Beitragsautoren* (Ausprägung 3000; inkl. Agenturmeldungen) selbst als Sprecher verschlüsselt.

Mehrmalige Nennung eines Sprechers/Akteurs sowie Zuordnung zu problembezogener Aussage der gleichen Ausprägung: Wird ein Sprecher/Akteur im Text mehrmals (d. h. ohne Änderungen seiner Rolle bzw. des institutionellen Kontextes) genannt und mehrfach mit einer problembezogenen Aussage der gleichen Ausprägung verknüpft, so wird diese Verknüpfung von Sprecher/Akteur und Aussage nur *einmal* codiert.

Mehrmalige Nennung eines Sprechers/Akteurs sowie Zuordnung zu unterschiedlichen problembezogenen Aussagen: Wird ein Sprecher/Akteur mehrmals genannt, aber mit problembezogenen Aussagen unterschiedlicher Ausprägung verknüpft, so wird der Sprecher/Akteur in einem solchen Artikel/Beitrag auch mehrmals als Sprecher in Verbindung mit der jeweiligen problembezogenen Aussage erfasst.

Einzelpersonen als öffentliche Stellvertreter für unabgeschlossene Personengruppen – Zuordnung zu problembezogener Aussage der gleichen Ausprägung: Falls in einem Artikel/Beitrag sowohl unabgeschlossene Personengruppen (z. B. „die CSU“) als auch Einzelpersonen (z. B. Alexander Dobrindt) vorkommen, die eindeutig der Personengruppe zugeordnet werden können bzw. im jeweiligen Artikel/Beitrag stellvertretend für diese sprechen, so werden – im Falle der Zuordnung zu einer problembezogenen Aussage der gleichen Ausprägung für Personengruppe und Einzelperson – die problembezogenen Aussagen zum Zwecke einer präziseren Identifikation *ausschließlich den Einzelpersonen* zugeordnet. Sofern in solchen Fällen *mehrere* unterschiedliche Einzelpersonen (im obigen Beispiel könnte neben Alexander Dobrindt und „der CSU“ auch Horst Seehofer auftreten) vorkommen, so sind die beiden Einzelpersonen separat zu verschlüsseln und jeweils der problembezogenen Aussage gleicher Ausprägung zuzuordnen. Diese Konventionen sind auch bei der Codierung der Kategorie SPRECH_EIN zu beachten.

Eine *Ausnahme* von diesen Regeln liegt nur dann vor, falls die unabgeschlossene Personengruppe als Sprecher/Akteur mit einer problembezogenen Aussage verbunden ist, die nicht auch von einer der Einzelpersonen vorgebracht wird. In diesem Fall ist die unabgeschlossene Personengruppe als separater Sprecher/Akteur mit einer eigenen problembezogenen Aussage zu verschlüsseln.

Gemeinsame Nennung von mehreren Sprechern/Akteuren und problembezogener Aussage der gleichen Ausprägung: Für den Fall, dass mehrere Sprecher/Akteure *gleichzeitig* mit einer problembezogenen Aussage der gleichen Ausprägung vorkommen (z. B. bei Formulierungen wie „Horst Seehofer und Angela Merkel sind sich in der Ansicht einig, dass…), so ist jeder angeführte Sprecher/Akteur *separat* zu verschlüsseln und der problembezogenen Aussage einzeln zuzuordnen.

Stilform ‚Kommentar, Kolumne, Glosse, Leitartikel‘: Bei dieser Stilform gilt für die Zuordnung zu problembezogenen Aussagen eine *Sonderregel*. Alle aufgeführten problembezogenen Aussagen werden ausschließlich dem Beitragsautor zugeordnet.

Problembezogene Aussagen in veröffentlichten Gesetzestexten und Originaldokumente: Im Falle von veröffentlichten Gesetzestexten oder Dokumenten werden diejenigen Personen als Sprecher/Akteur codiert, die die jeweiligen Unterlagen beschlossen, verabschiedet etc. haben.

Einzelpersonen und unabgeschlossene Personengruppen (SPRECH_EIN)

Mithilfe dieser Kategorie wird verschlüsselt, ob es sich bei den jeweiligen Sprechern/Akteuren um Einzelpersonen (z. B. Angela Merkel, Horst Seehofer) handelt oder ob problembezogene Aussagen unabgeschlossenen Personengruppen (z. B. „die CDU“, „die Regierung“, „die EU“, „die Menschen in Deutschland“, „die Bewohner der Stadt X“) zuzuordnen sind.

1 = Einzelperson

2 = Unabgeschlossene Personengruppe

Hinweis – Einzelpersonen als öffentliche Stellvertreter für unabgeschlossene Personengruppen – siehe auch die Vorbemerkungen zur Sprecher-/Akteurscodierung oben: Falls in einem Artikel/Beitrag sowohl unabgeschlossene Personengruppen (z. B. „die CSU“) als auch Einzelpersonen (z. B. Alexander Dobrindt) vorkommen, die eindeutig der Personengruppe zugeordnet werden können bzw. im jeweiligen Artikel/Beitrag stellvertretend für diese sprechen, so werden – im Falle der Zuordnung zu einer problembezogenen Aussage der gleichen Ausprägung für Personengruppe und Einzelperson – die problembezogenen Aussagen zum Zwecke einer präziseren Identifikation *ausschließlich den Einzelpersonen* zugeordnet. Sofern in solchen Fällen *mehrere* unterschiedliche Einzelpersonen (im obigen Beispiel: CSU, Alexander Dobrindt, Horst Seehofer) vorkommen, so sind die beiden Einzelpersonen separat zu verschlüsseln und jeweils der problembezogenen Aussage zuzuordnen.

Eine *Ausnahme* von diesen Regeln liegt nur dann vor, falls die unabgeschlossene Personengruppe als Sprecher/Akteur mit einer problembezogenen Aussage verbunden ist, die nicht auch von einer der Einzelpersonen vorgebracht wird. In diesem Fall ist die unabgeschlossene Personengruppe als separater Sprecher/Akteur mit einer eigenen problembezogenen Aussage zu verschlüsseln.

Hinweis – Beitragsautoren als Sprecher: Sofern einer oder mehrere Beitragsautoren selbst als Sprecher fungieren, ist immer die Ausprägung 1 (Einzelperson) zu codieren.

Hinweis – Nachrichtenagenturen: Bei Agenturmeldungen (angezeigt durch ein entsprechendes Kürzel wie „dpa“) werden Nachrichtenagenturen als Einzelpersonen gefasst.

Name des Sprechers/Akteurs (SPRECH_NAM)

Hier wird der im Text genannte Vor- und Nachname einer Einzelperson, in Sonderfällen die Namen von mehreren Einzelpersonen (getrennt durch ein Semikolon und in der Reihenfolge ihres ersten Auftretens im Originalbeitrag; siehe die Vorbemerkungen und Hinweise zur Sprecher-/Akteurscodierung), die Bezeichnung der unabgeschlossenen Personengruppe oder – sofern ersichtlich – der Vor- und Nachname des Beitragsautors bzw. der Beitragsautoren *offen* erfasst.

Hinweis – nicht näher definierte, unabgeschlossene Personengruppen: Sofern nicht näher definierte, unabgeschlossene Personengruppen vorkommen (Ausprägung 4000), wird in das offene Feld die im Artikel/Beitrag genutzte Umschreibung dieser Personengruppe eingetragen, z. B. „die Menschen aus Deutschland".

Hinweis – fehlende Namen oder Kürzel von journalistischen Beitragsautoren: Falls der Name von journalistischen Beitragsautoren (Ausprägung 3000) nicht zu erkennen ist, wird das Autorenkürzel oder – sofern dieses ebenfalls nicht angegeben ist – „Beitragsautor" in das offene Feld eingetragen.

Hinweis – mehrere journalistische Beitragsautoren: Falls mehrere journalistische Beitragsautoren gemeinsam einen Artikel/Beitrag verfassen, so sind alle Namen oder Kürzel – getrennt durch ein Semikolon und in der Reihenfolge ihrer Nennung im Originalbeitrag – hintereinander einzutragen.

Hinweis – ‚Name' bei Agenturmeldungen: Bei Agenturmeldungen wird das Kürzel der Nachrichtenagentur (z. B. dpa) als Name festgehalten.

Hinweis – mehrere Bezeichnungen für Personen: Sofern in einem Text mehrere Bezeichnungen für eine Person verwandt werden („Kanzlerin", „Angela Merkel", „Mutti"), so ist unter dieser Kategorie vornehmlich der Vor- und Nachname der Person zu verschlüsseln. Kommt der Vor- und Nachname nicht vor, so ist die institutionelle Rolle der Person festzuhalten. Nur dann, wenn ausschließlich Spitznamen oder ähnliche Umschreibungen vorkommen, sind diese an dieser Stelle offen zu erfassen.

Zuordnung von Sprechern/Akteuren zu Institutionen und Organisationen (SPRECH)

Sprecher und Akteure sollen mithilfe dieser Kategorie Institutionen oder Organisationen zugeordnet werden für die sie tätig sind bzw. für die sie öffentlich sprechen.

Hinweis – Oberkategorie „Politik“: Unterhalb der Oberkategorie „Politik“ ist zu differenzieren, in welcher Rolle bzw. in welchem institutionellen Kontext (Regierung, Parlament/Legislative, Parteifunktion) politische Akteure auftreten, z. B. ob die jeweilige Person in ihrer Funktion als Regierungsmitglied oder als Parteifunktionär spricht. Wenn *mehrere* Funktionen eines Sprechers/Akteurs aus der Oberkategorie „Politik“ genannt werden, sollte das *Staatsamt* der jeweiligen Person erfasst werden.

Hinweis – unabgeschlossene Personengruppen: Falls unabgeschlossene Personengruppen als Sprecher/Akteure vorkommen und sofern keine passenden, eigenständigen Ausprägungen vorliegen, sind Oberkategorien zu verschlüsseln. Beispiele: „die EU“ = Regierung → Europäische Ebene; „die deutsche Regierung“ oder „die Regierungskoalition“ = Regierung → Bundesebene; „der deutsche Staat“ = Verwaltung → Bundesebene; „NRW“ = Verwaltung → Landesebene; „die CDU“ = Parteifunktion → Bundesebene; „die NRW-CDU“ = Parteifunktion → Landesebene; „aus ungarischen Regierungskreisen“ = Regierung → Ausland; „die Politik“ = Politik. Im Falle von sehr allgemeinen, nicht näher definierten Personengruppen („die Menschen in Deutschland“, „die Bewohner der Stadt X“) wird die Ausprägung 4000 (Allgemeinheit als Sprecher) verschlüsselt.

Hinweis – Recherchen zur Identifikation von passenden Ausprägungen: Falls Recherchen zur korrekten Vergabe von Codes notwendig sind, so ist in Zweifelsfällen die *Selbstdarstellung* von Institutionen oder Organisationen auf deren Webseiten entscheidend. Beispiel: Das „Institut der deutschen Wirtschaft“ (IW Köln) könnte prinzipiell den Ausprägungen 2002 oder 2400 zugeordnet werden. Selbst stellt sich das IW Köln als „privates Wirtschaftsforschungsinstitut“ dar. Als Forschungsinstitut ist es der Ausprägung 2400 (Experten – Gutachter – Wissenschaft) zuzuordnen.

Hinweis – Darstellung von Sprechern/Akteuren in ehemaligen Funktionen: Im Falle von Darstellungen wie z. B. „In seiner früheren Funktion als Bundesinnenminister stand Thomas de Maizière für eine andere Migrationspolitik.“ ist darauf zu achten, ob problembezogene Aussagen im Text mit der früheren oder der heutigen Funktion (als „ehemaliger X“) von Spre-

chern/Akteuren verknüpft sind. Davon hängt ab, welche Ausprägung bei SPRECH verschlüsselt wird: Die ehemalige Funktion ist konkret zu verschlüsseln (im Beispiel also Ausprägung 1128), sofern Aussagen im Text eindeutig mit dieser früheren Funktion verbunden sind. Ansonsten wird Ausprägung 1400 codiert.

1000 POLITIK

1100 Rolle bzw. institutioneller Kontext: REGIERUNG (bzw. exekutive Äquivalente)

1110 Europäische Ebene (auch: Euro-Gruppe)

1111 EU-Kommission

1112 Europäischer Rat

1113 Rat der Europäischen Union (Ministerrat)

1114 Offizielle Sprecher europäische Top-Ebene (EU-Kommission, Europäischer Rat)

1120 Bundesebene

1121 Kanzler/in

1122 Vize-Kanzler/in, stellvertretende Regierungschefs

1123 Minister – Auswärtiges Amt

1124 Minister – BM Arbeit und Soziales

1125 Minister – BM Bildung und Forschung

1126 Minister – BM Familie, Senioren, Frauen, Jugend

1127 Minister – BM Finanzen

1128 Minister – BM Inneres

1129 Minister – BM Justiz und Verbraucherschutz

1130 Minister – BM Verteidigung

1131 Minister – BM Wirtschaft und Energie

1132 Minister – BM Wirtschaftliche Zusammenarbeit und Entwicklung

1133 Minister – Sonstige BM

1134 Offizielle Sprecher Bundesregierung

1135 Bundespräsident

1140 Landesebene

1141 Landesregierung/Staatsregierung/Senat

1142 Ministerpräsidenten/Regierender bzw. Erster Bürgermeister

1143 Landesminister/Staatsminister/Senatoren

1144 Offizielle Sprecher Landesregierung

1150 Kommunale Ebene

1151 Landrat

1152 Oberbürgermeister, Bürgermeister, Magistrat, Gemeindevorstand

1160 Ausland

1161 Staatsoberhäupter, Regierungschefs

1162 Minister

1163 Offizielle Sprecher ausländische Regierung

1200 Rolle bzw. institutioneller Kontext: PARLAMENT/LEGISLATIVE

Hinweis – unabgeschlossene Personengruppen im Bereich Parlament/Legislative: Für unabgeschlossene Personengruppen („die CDU-Fraktion", „der Rechtsausschuss") finden sich hier dementsprechende Ausprägungen (wie etwa Fraktion, Regierungsmehrheit, Opposition, Ausschuss). Diese sind der Codierung von Oberkategorien vorzuziehen. Im Falle von Fraktionen ist – sofern ersichtlich – auch die Parteizugehörigkeit zu verschlüsseln (siehe Kategorie SPRECH_PAR).

1210 Europäische Ebene

1211 Europäisches Parlament (EP)

1212 Präsident des EP

1213 Fraktion

1214 Fraktionsvorsitzende/r

1215 Fraktionsübergreifende Parlamentariergruppe

1216 Ausschuss

1217 Ausschussvorsitzende/r

1218 Einzelne Abgeordnete ohne genannte Funktion

1220 Bundesebene

1221 Bundestag

1222 Bundestagspräsident

1223 Regierungsmehrheit

1224 Opposition

1225 Bundesrat

1226 Fraktion (inkl. Landesgruppen innerhalb einzelner Parteifraktionen)

1227 Fraktionsvorsitzende/r (d. h. auch Dobrindt als Vorsitzender der CSU-Landesgruppe)

1228 Fraktionsübergreifende Parlamentariergruppe

1229 Ausschuss

1230 Ausschussvorsitzende/r

1231 sonstige Ausschüsse und Kommissionen (z. B. Enquete-Kommission, Vermittlungsausschuss, Gemeinsamer Ausschuss)

1232 Einzelne Abgeordnete ohne genannte Funktion

1240 Landesebene

1241 Landtag/Abgeordnetenhaus/Bürgerschaft

1242 Regierungsmehrheit

1243 Opposition

1250 Kommunale Ebene

1251 Landkreistag und Kreistag/Stadtrat/Gemeinderat

1260 Ausland

1261 Legislativ-parlamentarische Ebene Ausland

1300 Rolle bzw. institutioneller Kontext: PARTEIFUNKTION

1310 Europäische Ebene

1311 Parteivorsitzende

1312 Stellvertretender Parteivorsitzende

1313 Geschäftsführer bzw. Generalsekretär

1314 Sonstige Parteifunktionäre

1320 Bundesebene

1321 Parteivorsitzende

1322 Stellvertretender Parteivorsitzende

1323 Geschäftsführer bzw. Generalsekretär

1324 Sonstige Parteifunktionäre

1330 Landesebene

1331 Parteivorsitzende

1332 Stellvertretender Parteivorsitzende

1333 Geschäftsführer bzw. Generalsekretär

1334 Sonstige Parteifunktionäre

1340 Kommunale Ebene

1341 Vorsitzende

1342 Sonstige Parteifunktionäre

1350 Ausland

1351 Vorsitzende

1352 Sonstige Parteifunktionäre

1400 SONSTIGE SPRECHER/AKTEURE Politik national und international, z. B. bei Formulierungen wie „Innenpolitiker der CDU", bei denen der institutionelle Kontext sowie die Bezugsebene unklar bleiben sowie bei ehemaligen politischen Akteuren)

1500 VERWALTUNG

Hinweis – Verwaltungsorgane im Justiz- und Sicherheitsbereich: Verwaltungsorgane aus dem Justiz- und Sicherheitsbereich werden gesondert erfasst (siehe Code 1600).

Hinweis – Wahlbeamte: Wahlbeamte im deutschen Verwaltungssystem werden nicht der politischen, sondern der Verwaltungsebene zugeordnet (Ausprägung 1560).

1510 Europäische Ebene (hier nicht Grenzschutz-Agentur Frontex, siehe Code 1614)

1511 Verwaltungsorgane der Europäischen Kommission (Generaldirektionen und Dienste)

1512 Agenturen / Behörden / Ämter der EU

1513 Europäische Zentralbank

1520 Bundesebene (Hinweis: Beamtete sowie parlamentarische Staatssekretäre hier verorten)

1521 Bundeskanzleramt

1522 Presse- und Informationsamt der Bundesregierung

1523 Auswärtiges Amt (hier auch: Auswärtiger Dienst, Botschaften, ständige Vertretungen und Konsulate)

1524 Bundesministerium (BM) Arbeit und Soziales

1525 BM Bildung und Forschung

1526 BM Familie, Senioren, Frauen, Jugend

1527 BM Finanzen

1528 BM Inneres

1529 BM Justiz und Verbraucherschutz

1530 BM Verteidigung

1531 BM Wirtschaft

1532 BM Wirtschaftliche Zusammenarbeit und Entwicklung

1533 Sonstige BM

1534 Bundesamt für Migration und Flüchtlinge

1535 Bundesagentur für Arbeit

1536 Beauftragter der Bundesregierung für Menschenrechtspolitik und Humanitäre Hilfe (Menschenrechtsbeauftragter, angesiedelt im Auswärtigen Amt)

1537 Sonstige Ämter und Behörden (z. B. Bundeskartellamt, Bundesrechnungshof, Finanzverwaltung, Umweltbundesamt, Bundesverwaltungsamt etc.)

1540 Landesebene

Hinweis – Bezeichnungen von Landesministerien: Auf deutscher Landesebene gibt es keine einheitlichen Bezeichnungen für Ministerien. Aufgeführt sind hier Bezeichnungen von Ministerien mit inhaltlichem Bezug zu Flucht- und Asylmigration. Sollten in einem bestimmten Bundesland Ministerien für Themenbereiche zuständig sein, die hier mit unterschiedlichen Ausprägungen (Beispiel: Ministerium Wirtschaft, Arbeit und Wohnungsbau in Baden-Württemberg) versehen sind, so ist der Code (LM Wirtschaft, LM Arbeit, Soziales, Integration oder Sonstige LM) zu wählen, der thematisch am besten zu den fokussierten *Aussagen* des jeweiligen Sprechers/Akteurs passt.

1541 Staatskanzlei, Senatskanzlei

1542 Landesministerium (LM) Arbeit, Soziales, Integration

1543 LM Bildung, Forschung, Wissenschaft, Kultus

1544 LM Familie, Senioren, Frauen, Jugend, Gleichstellung

1545 LM Finanzen

1546 LM Inneres, Kommunales, Sport

1547 LM Justiz

1548 LM Wirtschaft

1549 Sonstige LM

1550 Landesämter und Behörden (z. B. Statistisches Landesamt, nicht: Landesämter für Verfassungsschutz oder Landeskriminalämter, siehe Code 1624)

1560 Kommunale Ebene, z. B. Regierungspräsidium, Landratsamt, Rathaus, Ordnungsamt, Kinder- und Jugendämter, Bürgeramt, Bürgerbüros, auch: Wahlbeamte

1570 Kommunale und stadtnahe Einrichtungen, z. B. Schulen, Wohnungsbaugesellschaften, Jugendzentren, sozialpädagogische Betreuungsangebote, Verkehrsbetriebe, Stadtwerke, Arbeitsvermittlung im Sinne von lokalen Geschäftsstellen und Job-Centern der Arbeitsagentur

1580 Ausländische Verwaltung, z. B. Ministerialverwaltung inkl. Staatssekretären, Ämter, Behörden etc.

1600 JUSTIZ UND SICHERHEITSORGANE

1610 Europäische Ebene

1611 Europäischer Gerichtshof

1612 Europol

1613 Eurojust

1614 Frontex (Grenzschutz-Agentur der EU)

1620 Deutschland

1621 Polizeien und Polizei-Organe auf Bundes-, Landes- und kommunaler Ebene

1622 Gerichte des Bundes und der Länder (auch: Amtsgerichte)

1623 Generalbundesanwalt, Staatsanwaltschaft

1624 Bundesamt und Landesämter für Verfassungsschutz

1625 Bundeskriminalamt und Landeskriminalämter

1626 Bundesnachrichtendienst

1627 Bundesamt für Justiz

1628 Rechtsanwälte

1629 Sonstige Organe aus dem Bereich Justiz und Sicherheit

1630 Ausland

1631 Rechtsprechung (ausländische Justiz)

1632 Rechtsdurchsetzung (ausländische Polizei)

1700 INTERNATIONALE STAATLICHE ZUSAMMENSCHLÜSSE bzw. INTERNATIONALE ORGANISATIONEN und ZUGEHÖRIGE ORGANE

1701 UN/UNO (hier u. a. auch: UNHCR, UN Sicherheitsrat, UN Menschenrechtsrat, Internationaler Gerichtshof, Internationale Arbeitsorganisation; nicht: UNO Flüchtlingshilfe, siehe „Interessenverbände“)

1702 OSZE

1703 WTO

1704 G7/G20

1705 OECD

1706 IWF

1709 Sonstige internationale staatliche Zusammenschlüsse bzw. Organisationen

1800 MILITÄR UND TERRORISTISCHE VEREINIGUNGEN

1801 Nationales Militär (z. B. Bundeswehr)

1802 Rebellen, Terrororganisationen, militante Extremisten

1803 Internationale Militärbündnisse (z. B. NATO, UN-Friedenstruppe/Blauhelme)

1809 Sonstige militärische Gruppe

1900 WIRTSCHAFT, z. B. Börse, private Banken, Unternehmen, nicht: staatliche oder supranationale Zentralbanken, diese fallen in den Bereich „Verwaltung“

2000 INTERESSENVERBÄNDE – SOZIALE BEWEGUNGEN – THINK TANKS – LOBBY-GRUPPEN – NGOs

2001 Menschenrechts-, Hilfs- und Nichtregierungsorganisationen sowie Vereine und Organisationen zur Unterstützung von Migranten (z. B. Brot für die Welt, Amnesty International, Ärzte ohne Grenzen, Human Rights Watch, Reporter ohne Grenzen, Vereine wie Pro Asyl, Amadeu Antonio Stiftung, UNO Flüchtlingshilfe)

2002 Interessengruppen Wirtschaft (Wirtschafts-, Arbeitgeber-, Bauernverbände wie BDI oder BDA, pro-wirtschaftliche Vereine, Stiftungen, Organisationen wie z. B. Initiative Neue Soziale Marktwirtschaft)

2003 Interessengruppen Soziales (Gewerkschaften wie verdi, DGB, IG Metall, auch: Betriebsräte, Vertreter von Polizeigewerkschaften; Hinweis: Wohlfahrtsorganisationen gesondert erfassen)

2004 Rechtspopulistische und rechtsextreme soziale Bewegungen (z. B. PEGIDA, Identitäre Bewegung)

2005 Linke und linksextreme soziale Bewegungen (No Border Netzwerk, Antifa, Anti-Globalisierung-Bündnisse)

2006 Migranten(selbst)organisationen (z. B. Türkische Gemeinde in Deutschland)

2007 Zusammenschlüsse von Interessengruppen (z. B. Europäischer Wirtschafts- und Sozialausschuss, Europäischer Ausschuss der Regionen)

2008 Sonstige Bürgergruppen (lokale Flüchtlingsinitiativen, Bürgerbewegungen, Bürgerallianzen)

2009 Sonstige(r) Interessenverband/Interessengemeinschaft (z. B. Umweltverbände, Deutscher Städte- und Gemeindebund)

2100 KIRCHE UND RELIGION, z. B. religiöse Oberhäupter, kirchliche/religiöse Amtsträger/Institutionen, religiöse Vereine, Verbände und Organisationen, nicht: Wohlfahrtsorganisationen)

2101 Christentum allgemein

2102 Katholische Kirche

2103 Evangelische Kirche

2104 Islam

2105 Judentum

2109 Sonstige Religionen

2200 WOHLFAHRTSORGANISATIONEN, z. B. Caritas, Diakonie, jüdische Zentralwohlfahrtsstelle, Deutsches Rotes Kreuz, Arbeiterwohlfahrt, Deutscher Paritätischer Wohlfahrtsverband

2300 MEDIZIN UND GESUNDHEIT, z. B. Selbsthilfegruppen, Krankenhaus, Rettungsdienst, Feuerwehr, Ärzte, Ärztekammer, Apotheken, Krankenkassen

2400 EXPERTEN – GUTACHTER – WISSENSCHAFT (nicht: Think Tanks, siehe Code 2000)

2500 MEDIEN UND JOURNALISTEN (nicht: Beitragsautor als Sprecher, siehe Code 3000)

2600 SPORT, z. B. Profi- und Amateur-Sport, Management, Verbände

2700 SONSTIGE EINZELPERSONEN

2701 Privatperson, einfache Bürger

2702 Ehrenamtlich Tätige

2703 Intellektuelle, Künstler

2704 Prominente (nicht: Politiker, Unternehmensvorstände, Sportler, Journalisten, etc.; nur zu codieren, falls keine zum Tätigkeitsbereich von prominenten Personen passende Ausprägung vorliegt)

2705 Migranten/Flüchtlinge/Asylbewerber

3000 BEITRAGSAUTOR(EN) ALS SPRECHER (inkl. Agenturmeldungen)

4000 ALLGEMEINHEIT ALS SPRECHER/AKTEUR, z. B. nicht näher definierte, unabgeschlossene Personengruppen wie „die Menschen in Deutschland“, „die Bewohner der Stadt X“ etc.

9000 SPRECHER/AKTEUR IST KEINER AUSPRÄGUNG ZUZUORDNEN

Parteizugehörigkeit von Sprechern/Akteuren (SPRECH_PAR)

Filter: Nur zu codieren, sofern ein Sprecher/Akteur, der a. als Einzelperson vorkommt (siehe SPRECH_EIN), b. unter SPRECH den Bereichen Politik oder Verwaltung zugeordnet wurde (d. h. Verschlüsselung von Codes im Zahlenbereich von 1000 bis 1580). Bei Sprecher/Akteuren, die diesen Bereichen nicht zuzuordnen sind, wird das Feld in der Codiermaske freigelassen. Erste Ausnahme von der Einzelperson-Bedingung stellen Parteien und Fraktionen dar. Kommen diese in der Form von unabgeschlossenen Personengruppen vor und wird die Parteizugehörigkeit im jeweiligen Text explizit genannt, so ist auch hier die Parteizugehörigkeit zu verschlüsseln. Eine zweite Ausnahme von der Einzelperson-Bedingung liegt vor, wenn bei unabgeschlossenen Personengruppen aus den Bereichen Politik und Verwaltung im Text explizit auf deren parteiliche Verortung verwiesen wird (Beispiele: „das grün regierte Friedrichshain", „die bayerische CSU-Regierung").

Hier wird die Parteizugehörigkeit von Sprechern und Akteuren – sofern erkenntlich – festgehalten. Bei Sprechern/Akteuren aus dem *Ausland* sind die jeweiligen zu verschlüsseln.

1 = SPD

2 = CDU

3 = CSU

4 = Die Grünen

5 = FDP

6 = Die Linke

7 = AfD

8 = NPD

9 = Sonstige Parteien (auch: Wählergemeinschaften)

10 = Parteilos (z. B. parteiloser Landrat/Bürgermeister)

11 = Sozialdemokratische, sozialistische Parteien

12 = Konservative, christdemokratische Parteien (USA: Republikanische Partei)

13 = Grüne, ökologische Parteien

14 = Liberale Parteien (USA: Demokratische Partei)

15 = Linke, linkspopulistische, kommunistische Parteien

16 = Rechtspopulistische/-extreme, europaskeptische, nationalistische, faschistische Parteien

17 = Sonstige Parteien

18 = Parteilos

99 = Parteizugehörigkeit nicht feststellbar

Literatur

Arlt, Dorothee, und Jens Wolling. 2016. The Refugees: Threatening or Beneficial? Exploring the Effects of Positive and Negative Attitudes and Communication on Hostile Media Perceptions. *Global Media Journal* 6 (1).

Beck, Ulrich, und Edgar Grande. *Cosmopolitan Europe.* Cambridge: Polity Press.

Benson, Rodney, und Tim Wood. 2015. Who Says What or Nothing at All? Speakers, Frames, and Frameless Quotes in Unauthorized Immigration News in the United States, Norway, and France. *American Behavioral Scientist* 59 (7): 802–821.

Blätte, Andreas. 2017. Multikulti ist tot? Lang lebe die Vielfalt! In *Regieren in der Einwanderungsgesellschaft: Impulse zur Integrationsdebatte aus Sicht der Regierungsforschung*, Hrsg. Christoph Bieber, Andreas Blätte, Karl-Rudolf Korte, und Niko Switek, 163–171. Studien der NRW School of Governance. Wiesbaden: Springer VS.

Budde, Nadine, Olaf Jandura, und Marco Dohle. 2018. Das Framing der Flüchtlingskrise in Parlament

Carter, Elisabeth. 2017. Party Ideology. In *The Populist Radical Right*, Hrsg. Cas Mudde, 28–67. London, New York: Routledge.

Eade, John, und Paolo Ruspini. 2014. Multicultural Models. In *An Introduction to Immigrant Incorporation Studies: European Perspectives*, Hrsg. Marco Martiniello und Jan Rath, 71–89. Amsterdam: Amsterdam University Press.

Eilders, Christiane, und Albrecht Lüter. 1998. *Methodenbericht zum Projekt: Die Stimme der Medien im politischen Prozess. Themen und Meinungen in Pressekommentaren*. WZB-Discussion Paper FS III 98-107.

Georgi, Fabian. 2015. Was ist linke Migrationspolitik? Warum globale Bewegungsfreiheit nicht nur ethisch geboten, sondern strategisch notwendig ist. *LuXemburg* (3): 110–115.

Georgi, Fabian. 2016. Offene Grenzen als Utopie und Realpolitik: Linke Strategien gegen Chauvinismus und soziale Konkurrenz. *LuXemburg* (1): 72–77.

Goedeke Tort, Maheba Nuria, Lars Guenther, und Georg Ruhrmann. 2016. Von kriminell bis willkommen: Wie die Herkunft über das mediale Framing von Einwanderern entscheidet. *Medien und Kommunikationswissenschaft* 64 (4): 497–517.

Grande, Edgar, und Hanspeter Kriesi. 2012. The Transformative Power of Globalization and the Structure of Political Conflict in Western Europe. In *Political Conflict in Western Europe*, Hrsg. Hanspeter Kriesi, Edgar Grande, Martin Dolezal, Marc Helbling, Dominic Höglinger, Swen Hutter, und Bruno Wüest, 3–35. Cambridge: Cambridge University Press.

Hainmueller, Jens, und Daniel J. Hopkins. 2014. Public Attitudes Toward Immigration. *Annual Review of Political Science* 17: 225–249.

Haller, Michael. 2017a. *Die „Flüchtlingskrise" in den Medien. Tagesaktueller Journalismus zwischen Meinung und Information*. OBS-Arbeitsheft, Bd. 93. Frankfurt: Otto Brenner Stiftung.

Haller, Michael. 2017b. Online-Teil zur „Haller-Studie": Die „Flüchtlingskrise" in den Medien. Methodische Hinweise. https://www.otto-brenner-stiftung.de/fileadmin/user_data/stiftung/02_Wissenschaftsportal/03_Publikationen/AH93_Fluechtlingskrise_Haller_Anhang.pdf. Zugegriffen: 19. November 2020.

Han, Petrus. 2010. *Soziologie der Migration*, 3. Aufl. Stuttgart: Lucius & Lucius.

Helbling, Marc. 2014. Framing Immigration in Western Europe. *Journal of Ethnic and Migration Studies* 40 (1): 21–41.

Hoesch, Kirsten. 2018. *Migration und Integration. Eine Einführung.* Wiesbaden: Springer VS.

Höglinger, Dominic, Bruno Wüest, und Marc Helbling. 2012. Culture versus Economy: The Framing of Public Debates over Issues related to Globalization. In *Political Conflict in Western Europe*, Hrsg. Hanspeter Kriesi, Edgar Grande, Martin Dolezal, Marc Helbling, Dominic Höglinger, Swen Hutter, und Bruno Wüest, 229–253. Cambridge: Cambridge University Press.

Joppke, Christian, und Ewa Morawska. 2003. Integrating Immigrants in Liberal Nation-States: Policies and Practices. In *Toward Assimilation and Citizenship: Immigrants in Liberal Nation-States*, Hrsg. Christian Joppke und Ewa Morawska, 1–37. New York: Palgrave Macmillan.
King, Natasha. 2016. *No Borders. The Politics of Immigration Control and Resistance.* London: Zed Books.
Kitschelt, Herbert. 1995. *The Radical Right in Western Europe. A Comparative Analysis.* Ann Arbor: University of Michigan Press.
Klos, Christian. 2013. Aufenthaltsrecht vor dem Infarkt: Ein rechtspolitisches Menetekel. In *Grenzüberschreitendes Recht - Crossing Frontiers: Festschrift für Kay Hailbronner*, Hrsg. Georg Jochum, Wolfgang Fritzemeyer, und Marcel Kau, 123–136. Heidelberg, München: C.F. Müller.
Koopmans, Ruud. 2017. *Assimilation oder Multikulturalismus? Bedingungen gelungener Integration.* Berlin: Lit.
Künzel, Mathias. 2007. Enthalten die Stellungnahmen der NPD-Fraktion im Plenum des sächsischen Landesparlamentes rechtsextremistische Elemente? Eine qualitative Inhaltsanalyse der Plenarprotokolle aus der 4. Legislaturperiode des Sächsischen Landtages, Technische Universität, Dresden. https://nbn-resolving.org/urn:nbn:de:bsz:14-qucosa-27521. Zugegriffen: 19. November 2020.
Küpper, Beate, Alexander Häusler, und Andreas Zick. 2016. Die Neue Rechte und die Verbreitung neurechter Einstellungen in der Bevölkerung. In *Gespaltene Mitte - Feindselige Zustände: Rechtsextreme Einstellungen in Deutschland 2016*, Hrsg. Friedrich-Ebert-Stiftung, 143–166. Bonn: Dietz.
Luft, Stefan. 2017. *Die Flüchtlingskrise. Ursachen, Konflikte, Folgen*, 2. Aufl. München: Beck.
Mediendienst Integration. 2018. Kriminalität in der Einwanderungsgesellschaft. https://mediendienst-integration.de/desintegration/kriminalitaet.html. Zugegriffen: 19. November 2020.
Mudde, Cas. 2017. Introduction to the Populist Radical Right. In *The Populist Radical Right*, Hrsg. Cas Mudde, 1–10. London, New York: Routledge.
Münkler, Herfried, und Marina Münkler. 2016. *Die neuen Deutschen. Ein Land vor seiner Zukunft.* Berlin: Rowohlt.
O'Brien, Peter. 2016. Transnationalism and Citizenship: Competing Normative Frames in Europe. In *Migration und Integration: Akzeptanz und Widerstand im transnationalen Nationalstaat*. Deutsche und internationale Perspektiven, Hrsg. Sandra Kostner, 289–320. Berlin: Lit.
Pfeiffer, Christian, Dirk Baier, und Sören Kliem. 2018. Zur Entwicklung der Gewalt in Deutschland: Schwerpunkte: Jugendliche und Flüchtlinge als Täter und Opfer. https://www.zhaw.ch/storage/shared/sozialearbeit/News/gutachten-entwicklung-gewalt-deutschland.pdf. Zugegriffen: 19. November 2020.
Philo, Greg, Emma Briant, und Pauline Donald. 2013. *Bad News for Refugees.* London: Pluto Press.
Roggeband, Conny, und Rens Vliegenthart. 2007. Divergent Framing: The Public Debate on Migration in the Dutch Parliament and Media, 1995-2004. *West European Politics* 30 (3): 524–548.
Ruhs, Martin, und Philip Martin. 2008. Numbers vs. Rights: Trade-Offs and Guest Worker Programs. *The International Migration Review* 42 (1): 249–265.
Schammann, Hannes. 2017. Reassessing the Opinion-Policy Gap: How PEGIDA and the AfD Relate to German Immigration Policies. In *Fortress Europe? Challenges and Failures of Migration and Asylum Policies*, Hrsg. Annette Jünemann, Nicolas Fromm, und Nikolas Scherer, 139–158. Wiesbaden: Springer VS.
Scheufele, Bertram, und Ines Engelmann. 2013. Die publizistische Vermittlung von Wertehorizonten der Parteien: Normatives Modell und empirische Befunde zum Value-Framing und News Bias der Qualitäts- und Boulevardpresse bei vier Bundestagswahlen. *Medien und Kommunikationswissenschaft* 61 (4): 532–550.

Stöss, Richard. 2010. *Rechtsextremismus im Wandel.* Berlin: Friedrich-Ebert-Stiftung.

Udris, Linards. 2011. *Politischer Extremismus und Radikalismus. Problematisierung und diskursive Gelegenheitsstrukturen in der öffentlichen Kommunikation der Deutschschweiz.* Wiesbaden: VS Verlag für Sozialwissenschaften.

van Gorp, Baldwin. 2005. Where is the Frame? Victims and Intruders in the Belgian Press Coverage of the Asylum Issue. *European Journal of Communication* 20 (4): 484–507.

Vertovec, Steven. 2007. Super-Diversity and its Implications. *Ethnic and Racial Studies* 30 (6): 1024–1054.

Voltmer, Katrin. 1997. Medien und Parteien im Wahlkampf: Die ideologischen Präferenzen der meinungsführenden Tageszeitungen im Bundestagwahlkampf 1990. *Rundfunk und Fernsehen* 45 (2): 173–193.

Voltmer, Katrin. 1999. *Medienqualität und Demokratie. Eine empirische Analyse publizistischer Informations- und Orientierungsleistungen in der Wahlkampfkommunikation.* Baden-Baden: Nomos.

Winkler, Jürgen R. 2001. Rechtsextremismus: Gegenstand - Erklärungsansätze - Grundprobleme. In *Rechtsextremismus in der Bundesrepublik Deutschland: Eine Bilanz*, Hrsg. Wilfried Schubarth und Richard Stöss, 38–68. Opladen: Leske + Budrich.

Zick, Andreas, Daniela Krause, und Beate Küpper. 2016. Rechtspopulistische und rechtsextreme Einstellungen in Deutschland. In *Gespaltene Mitte - Feindselige Zustände: Rechtsextreme Einstellungen in Deutschland 2016*, Hrsg. Friedrich-Ebert-Stiftung, 111–142. Bonn: Dietz.
